# Computational Assessment of the Cooperativity between RNA Binding Proteins and MicroRNAs in Transcript Decay

**DOI:** 10.1371/journal.pcbi.1003075

**Published:** 2013-05-30

**Authors:** Peng Jiang, Mona Singh, Hilary A. Coller

**Affiliations:** 1 Department of Computer Science, Princeton University, Princeton, New Jersey, United States of America; 2 Lewis-Sigler Institute for Integrative Genomics, Princeton University, Princeton, New Jersey, United States of America; 3 Department of Molecular Biology, Princeton University, Princeton, New Jersey, United States of America; University of Basel, Switzerland

## Abstract

Transcript degradation is a widespread and important mechanism for regulating protein abundance. Two major regulators of transcript degradation are RNA Binding Proteins (RBPs) and microRNAs (miRNAs). We computationally explored whether RBPs and miRNAs cooperate to promote transcript decay. We defined five RBP motifs based on the evolutionary conservation of their recognition sites in 3′UTRs as the binding motifs for Pumilio (PUM), U1A, Fox-1, Nova, and UAUUUAU. Recognition sites for some of these RBPs tended to localize at the end of long 3′UTRs. A specific group of miRNA recognition sites were enriched within 50 nts from the RBP recognition sites for PUM and UAUUUAU. The presence of both a PUM recognition site and a recognition site for preferentially co-occurring miRNAs was associated with faster decay of the associated transcripts. For PUM and its co-occurring miRNAs, binding of the RBP to its recognition sites was predicted to release nearby miRNA recognition sites from RNA secondary structures. The mammalian miRNAs that preferentially co-occur with PUM binding sites have recognition seeds that are reverse complements to the PUM recognition motif. Their binding sites have the potential to form hairpin secondary structures with proximal PUM binding sites that would normally limit RISC accessibility, but would be more accessible to miRNAs in response to the binding of PUM. In sum, our computational analyses suggest that a specific set of RBPs and miRNAs work together to affect transcript decay, with the rescue of miRNA recognition sites via RBP binding as one possible mechanism of cooperativity.

## Author Summary

Transcript degradation represents an important mechanism of regulation used in diverse biological processes, including during development to eliminate maternally inherited transcripts, in adult tissues to define cell lineages, and as part of signaling pathways to down-regulate unneeded transcripts. RNA binding proteins (RBPs) and microRNAs are two major classes of molecules utilized to degrade transcripts. Using computational methods, we analyzed the genomewide cooperativity between microRNA and RBP recognition sites. We observed cooperativity between Pumilio (PUM) and specific microRNAs that impacts transcript decay. Our analysis suggests that approximately seven mammalian microRNAs preferentially co-localize with PUM binding sites, and these microRNAs have recognition motifs that are reverse complements to the PUM recognition motif. Their binding sites are more likely to form RNA hairpin structures with proximal PUM recognition sites that would limit microRNA efficiency, but would be more accessible to microRNAs in response to the binding of PUM. These results indicate that rescuing microRNA recognition sites from hairpin structures may be an important role for PUM.

## Introduction

Transcript degradation is an important mechanism for regulating the levels of proteins in a time or space-dependent manner [1]. One mechanism through which transcript degradation can be controlled is via miRNAs, short RNAs approximately 21–23 nucleotides in length that regulate diverse biological processes [2], [3]. miRNAs are initially transcribed as pri-miRNAs, processed to form pre-miRNAs, which are hairpins of approximately 70–80 nucleotides, exported from the nucleus, and further processed to generate the final mature dsRNA [2]. Mature miRNAs are then loaded into the RISC complex, where they associate with target transcripts, resulting in transcript degradation and translation inhibition [4].

miRNAs generally bind their targets through complementary pairing in a short 7 bp seed sequence [5], [6]. There are likely other factors that also determine whether a miRNA will effectively target a particular recognition site, and some of these factors may be 3′UTR sequences that reside outside of the complementary sequence that the miRNAs bind. As an example, AU-rich sequences surrounding the miRNA binding sites have been reported to enhance the efficacy of miRNA-mediated mRNA decay [6]–[8]. The location of the recognition site at the 5′ or 3′ end of the 3′UTR, and especially far away from the center of long 3′UTRs, has also been associated with improved miRNA efficiency [6]. Thus, given a target transcript with a specific miRNA recognition site sequence, its decay efficiency is likely to be determined by a number of variables not all of which are currently well-understood.

Transcript degradation can also be regulated by RNA binding proteins (RBPs). These proteins can affect transcript stability by binding to recognition sequences within 3′UTRs. Some RBPs, for instance, AU rich element (ARE) binding proteins or Pumilio (PUM), increase the degradation of target transcripts [9]–[17]. Others, like the HuR family of ARE-binding proteins [18], cause stabilization of the targeted message. Several genomewide studies have suggested that RBPs and miRNAs may functionally interact [19]. Mukherjee and colleagues found that microRNA depletion had a less dramatic effect on sites at which the HuR binding protein could also bind, indicating that HuR was likely competing with microRNAs for binding sites and stabilizing the targeted transcript [20]. In another study, an analysis of gene expression changes after miRNA transfection revealed that U-rich motifs similar to HuD binding sequences were associated with transcript down-regulation [21]. Finally, immunoprecipitation with antibodies to the PUM protein followed by microarray analysis of surrounding RNA sequences revealed that miRNA binding sites are overrepresented in 3′UTR sequences within close proximity to PUM binding sites [22].

Specific instances in which RBPs enhance or inhibit the effectiveness of miRNAs have been experimentally verified. Competition between miRNAs and RBPs for the same sequence has been reported [23]–[25]. For example, down-regulation of the cationic amino acid transporter 1 (CAT-1) mRNA by miR-122 is inhibited by stress, and the de-repression requires binding of HuR to the 3′UTR [23]. As another example, the RBP CRD-BP binds to the coding region of TrCP1 mRNA and stabilizes it by competing with miR-183 and thus preventing miRNA-dependent processing [25]. miRNAs and RBPs have also been reported to cooperate. HuR and the miRNA *let-7* repress c-MYC expression though a mechanism that requires both HuR and *let-7* [26]. The *C. elegans* PUM homolog puf-9 is required for 3′UTR-mediated repression of the *let-7* target hbl-1 [27]. In *Drosophila*, an association between the RBP dFXR and RISC is required for efficient RNA interference [28]. As a final example, an AU-rich motif located upstream of the miR-223 binding site in the 3′UTR of RhoB has been reported to enhance miRNA function [8].

One specific mechanism through which the Pumilio RNA binding protein has been proposed to modulate miRNA function is by binding to sequences that can hybridize with miRNA recognition sites and thereby make them more accessible for the RISC complex. Binding of PUM to the 3′UTR of the cyclin-dependent kinase inhibitor p27^Kip1^ has been reported to cause a local change in structure that promotes p27^Kip1^ repression by miR-221/miR-222 [29]. Another study demonstrated that binding of PUM facilitated miR-503 regulation of the E2F3 3′UTR [30]. The authors hypothesized PUM binding was able to relax the 3′UTR secondary structure elements that would otherwise block miR-503 binding sites. A final study on the pyrimidine-tract-binding (PTB) protein proposed that PTB binding can modulate the secondary structure of the GNPDA1 3′UTR to facilitate let-7b binding [31].

We hypothesized that miRNAs and RBPs might cooperate to facilitate transcript decay more extensively than had been realized. Using computational models, we systematically explored RBP-miRNA interactions within human and mouse 3′UTRs and discovered that RBP recognition sites co-occur with subsets of miRNA recognition sites. Our analyses revealed that PUM is likely to cooperate with specific miRNAs to promote decay. Moreover, we found that a subset of miRNAs that co-occur with PUM recognition sites have recognition seed sequences that are the reverse complements of the PUM recognition motif, and thus, may form hairpin secondary structures that would be disrupted by PUM binding. Based on our computational analysis, we discovered seven miRNAs in human and five in mouse that followed this pattern. Approximately 4% of the target sites for these miRNAs colocalize with PUM sites in a pattern that would have the potential for miRNA binding site rescue.

## Results

### RBP and miRNA recognition motif selection

**Figure 1:**
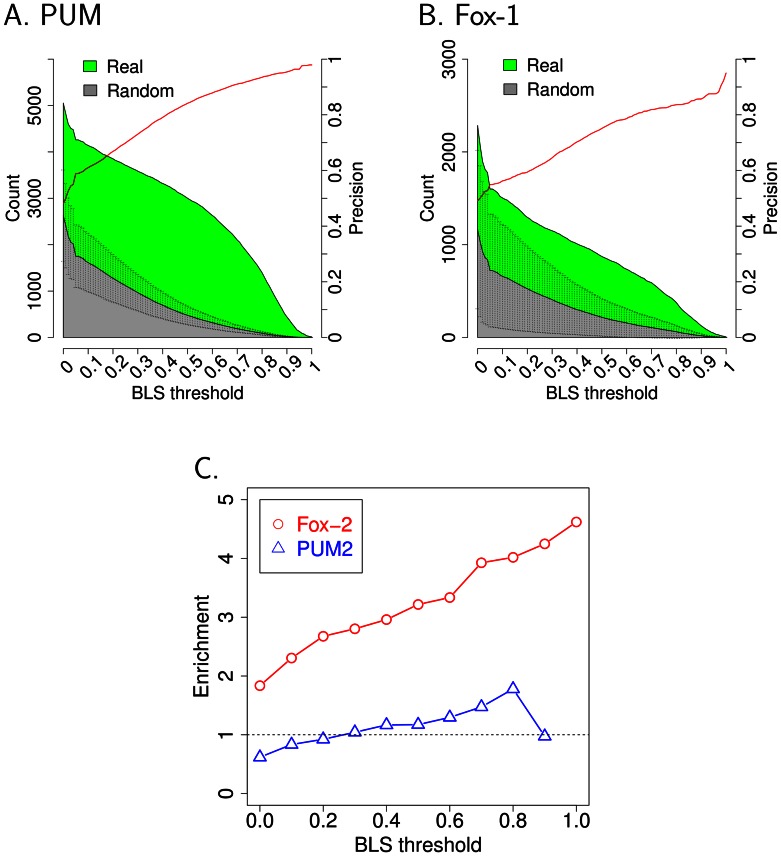
Identifying RBPs with binding sites evolutionarily conserved in 3′UTRs. (A, B) For each instance of a putative RBP recognition motif site in 3′UTRs, the Branch Length Score (BLS) was determined based on multiple genome alignments. The number of motifs at different levels of conservation (BLS) is plotted. The area below the curve for the true RBP is shaded green. The frequency with which randomly shuffled motifs were present in the genome is indicated in gray according to the y-axis on the left. Error bars indicate the standard deviation for the different shuffled versions of the motif. The precision ratio (1-[The average number of matches of shuffled motifs]/[The number of matches of the canonical motif]) is indicated by the red line according to the y-axis on the right. (C) CLIP-Seq binding regions for Fox-2 were mapped on human 3′UTRs [47]. At each conservation BLS threshold, an enrichment ratio was determined by comparing the density of binding sites within the CLIP region versus outside the CLIP region for the Fox-1 and Fox-2 binding motif UGCAUGU [46]. The BLS threshold is shown on the X-axis and enrichment is shown on Y axis. As a control, enrichment of the Fox-1 and Fox-2 motif in PUM2 Par-CLIP sequences was also plotted [37].

We performed a literature search and identified 15 instances in which an RBP and its putative recognition motif were reported [13], [32]–[37] (Figure S1). We reasoned that true RBP recognition motifs that are functional in 3′UTRs would be present more frequently than expected by chance, especially at high levels of evolutionary conservation. Using a method adapted from Kellis and colleagues [38], we found 5 out of the 15 RBPs had significantly increased conservation frequencies compared to their shuffled control motifs (Figure 1A, B and Figure S2). All five of these motifs have been demonstrated to be present in 3′UTRs by previous studies. The motifs are recognition motifs for the transcript decay factors PUM (UGUANAUA) [35], the Fox-1 family of proteins associated with splicing (UGCAUGU) [39]–[41], U1A (AUUGCAC)(a component of the snRNP complex) [42], [43], and Nova (YCAUUUCAY) [36], and the AU-rich element (ARE) UAUUUAU, which is bound by many different ARE binding proteins [9], [44].

For the PUM recognition motif, for instance, there is a large increase in the number of observed recognition sites compared with the number expected based on shuffled controls (Figure 1A). In contrast, U2B is reported to bind the sequence AUUGCAG [45], however, its putative binding sites were present a comparable number of times in 3′UTRs compared with shuffled versions of the motif at all levels of evolutionary conservation (Figure S2D). U2B and the nine other such RBPs were therefore not included in our subsequent analyses.

One example of a RBP motif that passed our threshold was the Fox-1 family binding site (UGCAUGU), which represents a family of RBPs that are well-conserved in metazoans. In mammals, there are three members of the Fox-1 family, Fox-1, Fox-2 and Fox-3 [46]. The Fox-1 RBP family recognizes sites with a consensus sequence of UGCAUGU [39] and matches to this sequence were consistently present in 3′UTRs at a higher frequency than shuffled controls (Figure 1B). This was somewhat unexpected because Fox-1 family RBPs are generally considered to be splicing factors [46]. To further confirm that the recognition sites on 3′UTRs that we designated as a Fox-1 family binding sites are bound by Fox-1 family RBPs, we analyzed 34,111 non-overlapping regions on human 3′UTRs identified in a previous study of Fox-2-associated sequences using next generation sequencing [47]. As a member of the Fox-1 RBP family, Fox-2 also binds UGCAUGU, so we compared the density of Fox-1 family motifs within the immunoprecipitated 3′UTR sequences with the density in 3′UTRs outside the immunoprecipitated sequences. The enrichment for the Fox-1 family motif increased monotonically with an increasing conservation threshold, from twice as frequent for all binding sites to 4 times more frequent when requiring perfect conservation through all placental mammals (Figure 1C). As a control, we didn't observe a significant enrichment for the Fox-1 motif within sequences immunoprecipitated with antibodies to PUM2 [37] (Figure 1C). We conclude that the computational approach that we are using to define RBPs that bind 3′UTRs is consistent with experimental data, and that members of the Fox-1 family likely do bind 3′UTRs.

### RBP motifs tend to localize to the end of long 3′UTRs

**Figure 2:**
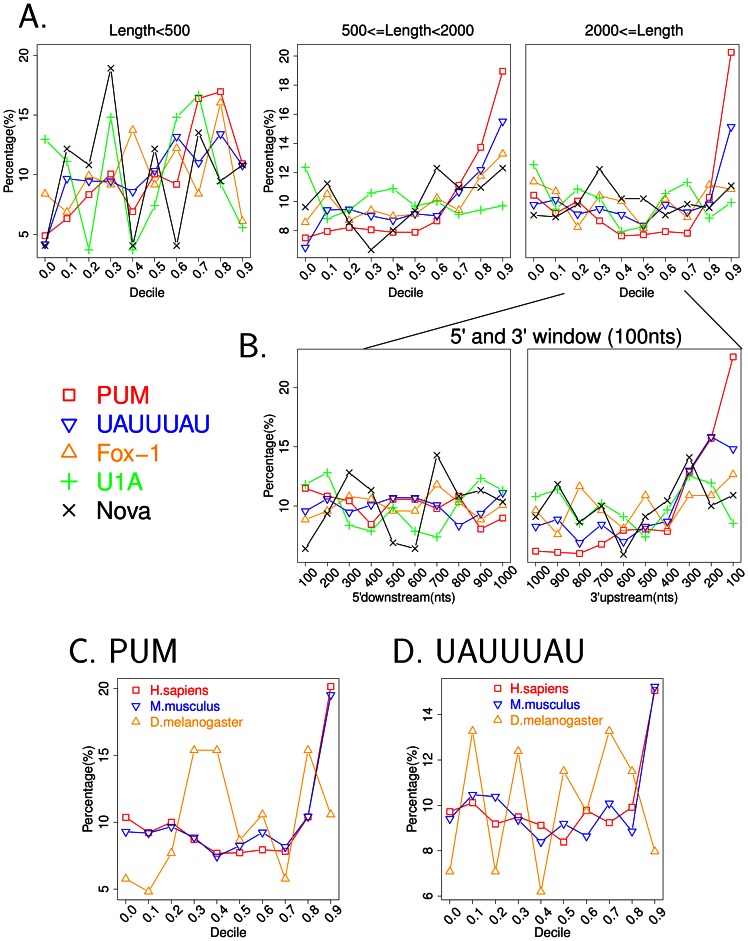
RBP motifs tend to localize to the end of long 3′UTRs. (A) All human 3′UTRs were classified into three length categories: smaller than 500 nts, longer than 2000 nts, or between 500 and 2000 nts. Each 3′UTR was equally divided into ten bins, numbered from 0.0 to 0.9. Within each length category, the percentage of RBP recognition sites in each bin was plotted. (B) For 3′UTRs longer than 2000 nts, ten 100-nt-windows from the 5′ start towards downstream and the 3′ end towards upstream were analyzed. The percentage of RBP recognition sites in each window was plotted. (C, D) Human (*Home sapiens*), mouse (*Mus musculus*) and fly (*Drosophila melanogaster*) (but not the worm *Caenorhabditis elegans*) contain 3′UTRs longer than 2000 nts. The localization patterns of PUM and UAUUUAU recognitions sites were plotted in ten bins for 3′UTRs longer than 2000 nts.

Previous analyses showed human miRNA recognition motifs tend to localize at the 5′ beginning or 3′ end of long 3′UTRs [48], [49]. For the five RBP recognition motifs included in this study, we investigated the localization of the associated RBP binding sites along 3′UTRs. We first classified the human 3′UTRs into three length categories: 3′UTRs with length <500 nts (6622 transcripts), 3′UTRs with length  = >500 nts and <2000 nts (7385 transcripts) and 3′UTRs with length > = 2000 nts (3759 transcripts). Within each length category, we divided 3′UTRs into 10 equal parts and counted the percentage of motif occurrences in each of the 10 bins. We observed that for RBP motifs PUM and UAUUUAU, the number of recognition sites is highest at the very end of the 3′UTRs longer than 500 nts (Figure 2A). For 3′UTRs longer than 2000 nts, we created ten 100-nt-windows from the 5′ beginning and 3′ end of the full UTR and counted the percentage of RBP motifs found in each window. The number of RBP motifs PUM and UAUUUAU was highest in windows located 100 nts and 200 nts from the end of the 3′UTRs (Figure 2B).

For RBP motifs such as PUM and UAUUUAU with high AU content, their preferential distribution at the very end of 3′UTRs could, in principle, reflect the higher AU-content at the end of long 3′UTRs. We analyzed the fraction of AU base pairs in different deciles of 3′UTRs and found that 3′UTRs tend to have high AU-content at the 3′ end region in human, mouse, fly and worm (Figure S3). In order to control for AU-content, we generated shuffled control motifs that have the same base pair composition as the initial motif for each RBP. We compared the percentage of RBP recognition sites in each 3′UTR bin with the average from all shuffled RBP motifs in the same bin (Figure S4). In the human genome, PUM recognition sites (binomial test *p*-value = 2.24E-23) and UAUUUAU (binomial test *p*-value = 6.57E-3) were significantly more frequent at the very 3′ end of 3′UTRs, even after correcting for the high AU content in this region of 3′UTRs (Figure S4A, B).

Having discovered that certain RBP recognition motifs are enriched at the 3′ ends of long 3′UTRs in human, we then asked whether this localization pattern is present in other species as well. PUM is part of a well-conserved family of PUF proteins [15], [50]. There are PUM proteins that bind the consensus sequence UGUANAUA in human [35], mouse [51], fly [52] and worm [53]. UAUUUAU is also a binding motif for RBPs in human, mouse [54], fly [55] and worm [56]. We analyzed the localization of the PUM and UAUUUAU consensus sequence within 3′UTRs in these four species and discovered that the preference for the 3′ most region of long 3′UTRs exists in human and mouse, but not fly and worm (Figure 2C, D for 3′UTRs longer than 2000 nts and Figure S5 for 3′UTRs shorter than 2000 nts but longer than 500 nts). For mouse, we also determined the extent to which AU content can explain the enrichment for PUM and UAUUUAU at the 3′ end of longer 3′UTRs. In a pattern similar to that observed in human, PUM strongly localized to the most 3′ decile of mouse 3′UTRs compared to shuffled control motifs (Figure S4C, binomial test *p*-value = 1.95E-9). However, UAUUUAU was present in a similar percentage of 3′UTRs compared to shuffled control motifs with the same AU content (Figure S4D), even though it is highest in the most 3′ decile. Thus, for mouse 3′UTRs, both PUM and UAUUUAU are enriched at the very end of 3′UTRs, but the UAUUUAU enrichment is likely explained by the high AU-content at the end of mouse 3′UTRs.

### Recognition sites for RBPs and specific miRNAs colocalize

We then asked whether the recognition sites of RBPs and miRNAs tend to be present close to each other on the same transcripts, as previous studies have reported that RBPs and miRNAs that functionally interact are often located close to each other [8], [26], [27]. For each pair of RBP and miRNA, we counted the number of neighboring RBP and miRNA recognition sites within 50 nts. As a control, we shuffled the identities of predicted miRNA recognition sites, while keeping their positions intact. An empirical *p*-value was calculated by comparing the observed number of neighboring RBP and miRNA recognition sites within 50 nts with the number of neighboring sites when the miRNA identities were randomized. For each RBP, miRNAs were classified as “interacting miRNAs” if they had a false discovery rate (FDR) less than 0.05 as determined by the Benjamini Hochberg procedure [57].

**Figure 3:**
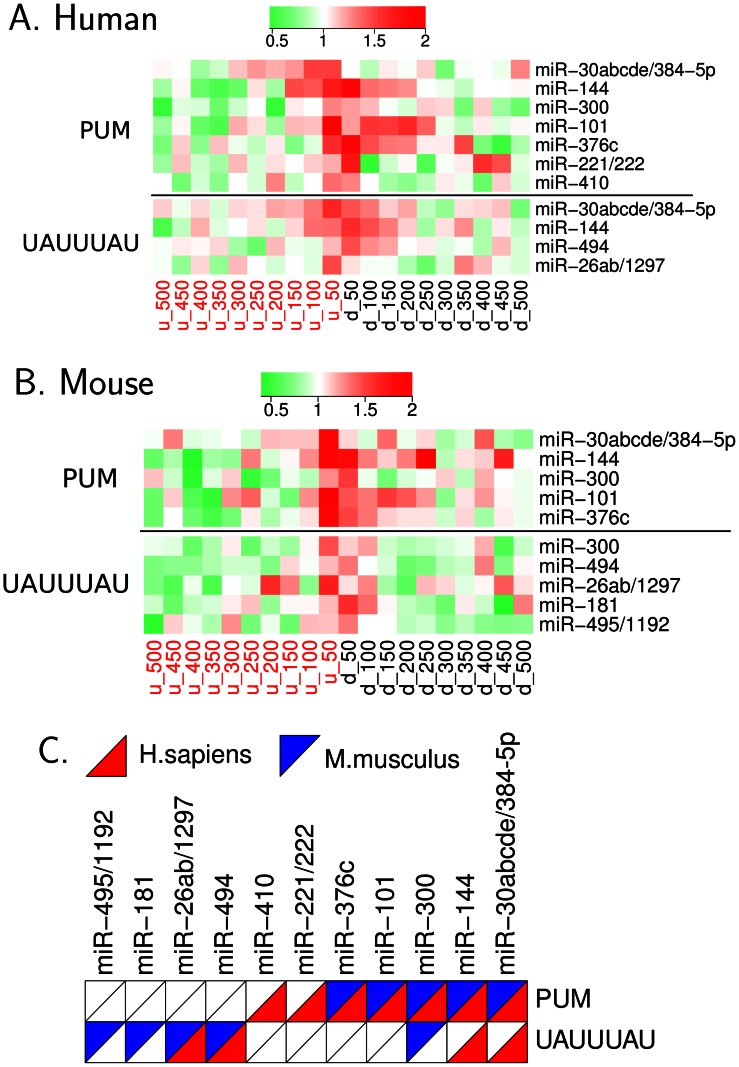
The recognition sites of RBP and specific miRNAs colocalize. For each RBP, the set of miRNAs with recognition sites that co-localize with the analyzed RBP with an FDR< = 0.05 are shown. The number of neighboring miRNA sites in ten 50 nt windows up- or down-stream of the RBP recognition site were compared to the number when the miRNA identities were shuffled. For each window, the ratio was determined as (number of miRNA sites)/(expected number based on shuffling). The miRNA site ratios were visualized in heatmap format with red indicating a high ratio and green indicating a low ratio. (A) Human miRNAs. (B) Mouse miRNAs. (C) A pairwise matrix between RBPs and interacting miRNAs is shown (FDR< = 0.05). In each cell, a red upward-sloping triangle is used to indicate colocalization in human and a blue downward-sloping triangle is used for mouse.

Among the five RBPs investigated, only PUM and UAUUUAU have miRNAs that are more abundant than expected within 50 nts of the RBP recognition site using this procedure (Table S1). For ten 50-nt windows upstream and downstream from RBP recognition sites, we plotted the ratio of the observed number of miRNA recognition sites to the expected number of sites, as estimated by randomly shuffling miRNA site identities (Figure 3A). As expected, for interacting miRNAs, the ratio of observed to expected events is high around the RBP sites, and is lower in more distant windows.

We performed a similar analysis to determine interacting miRNAs for the same RBPs in mouse and discovered that there are miRNAs that colocalize with the PUM recognition site or UAUUUAU in mouse 3′ UTRs (Figure 3B). Some miRNAs have recognition sites that co-localize with PUM and UAUUUAU in both species (Figure 3C). For example, five of the seven miRNAs identified as PUM-interacting miRNAs in the human genome are also PUM-interacting miRNAs in mouse.

For the interacting miRNAs, we calculated the percentage of all miRNA recognition sites that are located within 50 nts from the sites of their preferentially co-localized RBPs (Figure S6). For both PUM and UAUUUAU, the fraction of their interacting miRNA binding sites that are found proximal to RBP sites is around 4%. As expected, a smaller fraction of binding sites are proximal to the RBP recognition sites for non-interacting miRNAs in both human and mouse (Figure S6).

We further tested whether PUM and its predicted interacting miRNAs are enriched in experimental data in which the binding sites for both PUM2 and AGO were experimentally profiled in HEK293T cells by Par-CLIP (Photoactivatable Ribonucleoside Enhanced Crosslinking and Immunoprecipitation) [37]. We restricted the predicted PUM and miRNA recognition sites to only those identified by Par-CLIP analysis of PUM2 and AGO and counted the number of miRNA sites within 50 nts of PUM recognition sites. To define the background expectation, we permuted the identities of miRNA sites across chromosomes and counted the number of neighboring sites after restricting our analysis to sites within Par-CLIP regions. For each miRNA, an enrichment ratio was calculated as (the true number of neighboring sites)/(the average number of neighboring sites from 10000 shuffles). The interacting miRNAs showed significantly higher enrichment ratios than non-interacting miRNAs (Figure S7A).

We also recognized that not all miRNAs are expressed in all cells. To address this issue, the interacting miRNAs were further classified based on the sequence read abundance in HEK293T cells [37]. Expressed miRNAs, which were defined as the miRNAs with the top 25% read frequency, showed significantly higher enrichment ratios than non-expressed miRNAs (Figure S7B). Thus, the set of interacting miRNAs we predicted based on computational analysis of the genome sequence are also enriched based on Par-CLIP experimental data, and this enrichment shows the expected dependency on cell type-specific miRNA expression.

We also assessed the possible effects of AU content on co-localization of miRNA and RBP recognition sites. AU content has been reported to affect miRNA site effectiveness [6]. Indeed, the recognition motifs for PUM and UAUUUAU and their co-localizing miRNA recognition seeds tend to be AU-rich (Table S2). To ensure that the co-occurrence observed between miRNAs and RBPs was not caused exclusively by the high AU composition of these motifs and their colocalization in AU-rich regions of 3′UTR, we evaluated shuffled RBP motifs with the same AU composition (Methods). For the two RBPs with co-occurring miRNAs, miRNA recognition motifs exhibited enrichment around true RBP recognition motifs compared to shuffled RBP control motifs generated to preserve AU content in the windows 50 nts up- or down-stream of the RBP (Figure S8). The signal remained strong for PUM in both mouse and human, but was weaker for UAUUUAU after this correction. Thus, the RBP-miRNA co-localization that we observed, especially for PUM, cannot be explained simply by the AU composition of the recognition sites.

Using the same procedure as for human and mouse, no interacting miRNAs were predicted for fly and worm. We also determined the enrichment of miRNA recognition site density around each RBP site compared to the overall miRNA site density across all 3′UTRs with a RBP or miRNA recognition site. With this analysis, enrichment near the RBP recognition site for two RBP motifs was higher in human and mouse than the other two organisms (Table S3). However, since the quality of the 3′UTR annotations or the miRNA family member annotations may differ across organisms, more research will be needed to determine whether RBP-miRNA interactions are prevalent or limited to specific species.

### PUM and miRNAs cooperate to affect mRNA decay

We next examined the functional effect of RBPs and miRNAs on transcript decay using three genome-wide mRNA half-life datasets. These datasets are genomewide measurements of mRNA half-lives in human B cells and mouse fibroblasts [58] and mRNA decay rates in human HepG2 cells [59]. We determined the median half-life or decay rate for the set of transcripts that contain each of the recognition sites of interest. Transcripts containing a 3′UTR PUM or UAUUUAU site decayed faster than transcripts containing shuffled RBP motifs in all datasets (Figure S9). The presence of Fox-1, U1A or Nova recognition sites was not consistently associated with faster decay.

Having determined that for certain pairs of RBPs and miRNAs, their binding sites are frequently present in 3′UTRs in close proximity (Figure 3), we set out to further dissect the cooperativity between RBPs and miRNAs in mediating transcript decay. For each RBP, we divided all transcripts into four categories: “Int-proximal”: transcripts containing RBP recognition sites, and within 50 nts, a miRNA recognition site for one of the interacting miRNAs for that RBP; “Int-distant”: transcripts containing RBP recognition sites and miRNA recognition sites for one of the interacting miRNAs for that RBP, but none of the miRNA recognition sites and RBP recognition sites are within 50 nts of each other; “Nonint-proximal”: transcripts containing RBP recognition sites with at least one miRNA recognition site within 50 nts, but the miRNA is not an interacting miRNA for that RBP; “Nonint-distant”: transcripts containing RBP recognition sites with at least one non-interacting miRNA recognition site, but none of the RBP recognition sites and miRNA recognition sites are within 50 nts of each other.

**Figure 4:**
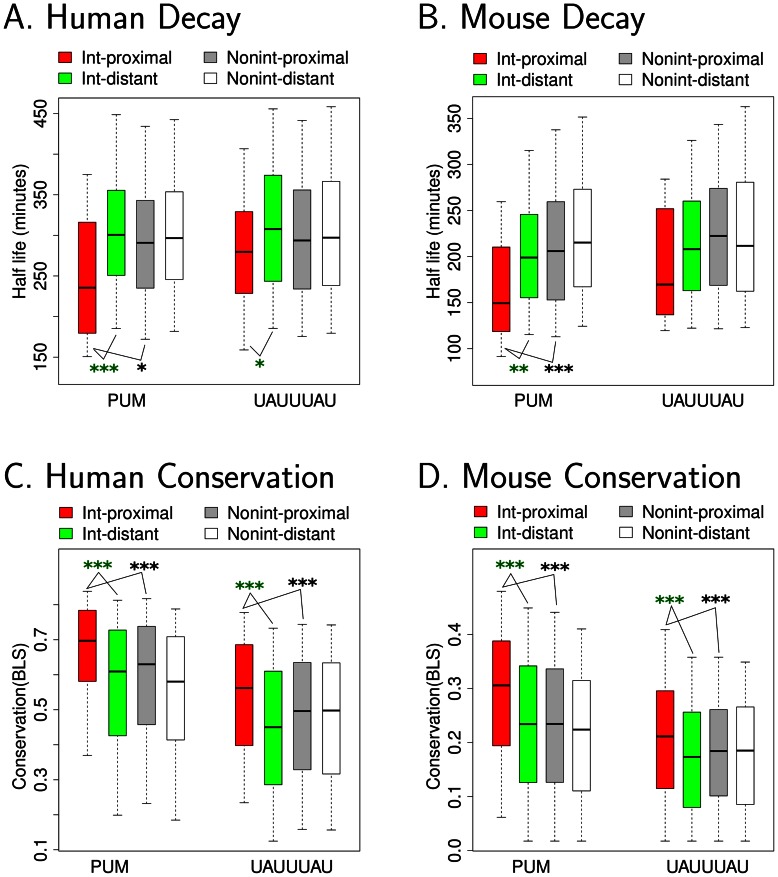
Pumilio recognition sites promote decay more effectively and are better conserved when present with interacting miRNAs. Transcripts with a specific RBP recognition site were divided into four groups. Group “Int-proximal” contained transcripts with at least one RBP site and its interacting miRNA recognition site within 50 nts. Group “Int-distant” contained transcripts with both a RBP recognition site and a recognition site for its interacting miRNA, but no pair of RBP-miRNA site is within 50 nts. Group “Nonint-proximal” and “Nonint-distant” were similar to group “Int-proximal” or “Int-distant” except non-interacting miRNAs (not predicted in Figure 3) were analyzed. For each group of transcripts, the half lives (or conservation scores) were ranked and the (25%, 75%) range of the data were extracted and plotted with box-plots for visualization. The bottom and top of the box are the 25th and 75th percentiles (the inter-quartile range). Whiskers on the top and bottom represent the maximum and minimum data points within the range represented by 1.5 times the inter-quartile range. For each RBP, asterisks represent comparisons of half-life (or conservation score) between “Int-proximal” and “Nonint-proximal” or between “Int-proximal” and “Int-distant” by Wilcoxon test on the full range of data. One asterisk indicates *p*<0.05, two asterisks indicate *p*<0.01, and three asterisks indicate *p*<0.001. (A, B) Half-lives for mRNAs are plotted for human and mouse [58]. (C, D) Conservation BLS scores for RBP recognition sites are plotted.

For each RBP, mRNA half-life values or decay rates determined experimentally by Friedel [58] and Yang [59] were considered for all transcripts in each of the four classes of transcripts defined above. For each mRNA decay dataset, data from small RNA sequencing experiments in the same cell line were used to define the set of miRNAs expressed, and only those miRNAs in the top 25% most sequenced miRNAs were considered for further analysis [60]–[62]. For PUM, the presence of nearby interacting miRNA recognition sites, but not distant miRNA sites or nearby non-interacting miRNA sites, consistently increased the decay rate in both human B cells and mouse fibroblasts [58] (Figure 4A, B and Table S4). Similar results were also observed in an independently derived human mRNA decay rate dataset [59] (Figure S10A). Moreover for transcripts with recognition sites for PUM and its interacting miRNAs within 50 nts, expressed miRNAs promote decay consistently faster than non-expressed miRNAs (Figure S11).

We also tested whether the more rapid decay observed in transcripts with recognition sites for both PUM and miRNAs was a consequence of the high AU-content of the PUM recognition sites and the recognition sites of its interacting miRNAs. We utilized shuffled control motifs of PUM and miRNAs that have the same AU-content as the real motif. We established three groups of transcripts according to the presence of PUM and miRNA recognition sites on 3′UTRs: (Real), real RBP recognition sites and real recognition sites for interacting miRNAs within 50 nts; (miR_control), real RBP recognition sites and sites for shuffled interacting miRNA motifs within 50 nts; and (RBP_control), shuffled RBP motif sites and real interacting miRNA sites within 50 nts. We observed that transcripts with real PUM and interacting miRNA recognition sites have consistently shorter half-lives compared to transcripts in the two other control groups (Figure S12). Thus, the more rapid decay rate observed in 3′UTRs with interacting PUM and miRNA recognition sites is not simply a consequence of the high AU content of recognition motifs of PUM and its interacting miRNAs.

For UAUUUAU, transcripts with both UAUUUAU sites and recognition sites of its interacting miRNAs in close proximity tend to have shorter mRNA half lives than transcripts in other groups (Figure 4A, B, Figure S10, S11 and S12). However, the effect is less strong than the effect observed for PUM.

In addition to analyzing mRNA decay, we also extended our observations to evolutionary conservation. We discovered that for both PUM and UAUUUAU, recognition sites that are located within 50 bps of an interacting miRNA are better conserved than recognition sites located more than 50 bps from an interacting miRNA or within 50 bps of a non-interacting miRNA in both human and mouse (Figure 4C, D, Figure S10B and Table S5).

We also ran Gene Ontology enrichment analysis for human genes with colocalized PUM and interacting miRNA recognition sites in their 3′UTRs. We found GO categories related to transcriptional regulation were enriched (Table S6). Thus, it is possible that the synergistic effects of PUM and miRNAs on mRNA decay rate will subsequently affect the initiation of transcription for genes.

### PUM rescues recognition site accessibility for PUM-interacting miRNAs

We further investigated why a specific group of miRNA recognition sites tend to be localized proximal to PUM recognition sites and promote decay. Previous studies have reported that for miR-221/222 and miR-410, PUM can alleviate the constraints of RNA secondary structure and make miRNA binding sites more accessible to the RISC complex [29], [63]. We hypothesized that the genome-wide co-occurrence of PUM and a specific set of miRNAs is related to the ability of PUM to rescue miRNA recognition site accessibility.

To address this issue on a genome-wide scale, we used a computational approach to estimate the frequency of RBP regulation of local 3′UTR secondary structure. For each pair of neighboring RBP-miRNA recognition sites, we determined the number of base pairs of miRNA recognition site that RBP binding can rescue from pairing with other nucleotides within the 3′UTR as estimated by RNAfold (Methods) [63]–[65]. As an example, when we used RNAfold to determine the secondary structure for the p27^Kip1^ 3′UTR, we discovered that 6 out of 7 base pairs of the miR-221/222 recognition seed site were hybridized to other nucleotides and therefore inaccessible due to the sequence's secondary structure. When we simulated PUM binding by converting all of the bases in the PUM recognition sites to N's, and thus made them unavailable to hybridize to other bases in the sequence, 0 base pairs of the miR-221/222 seed site were blocked. We calculate the amount of miRNA site rescue as 6−0 = 6.

**Figure 5:**
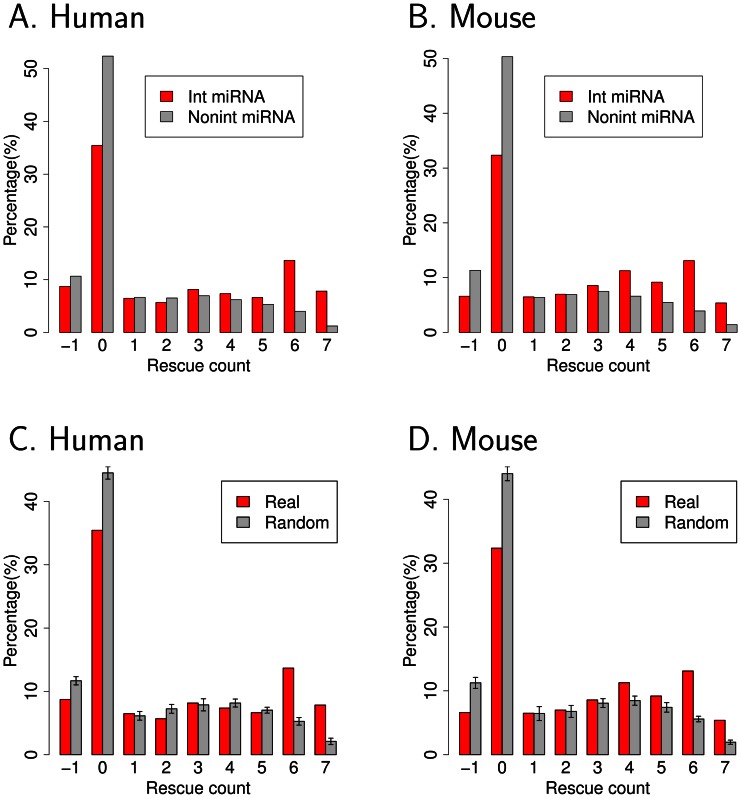
PUM rescues nucleotides in neighboring interacting miRNA recognition sites. (A, B) For each PUM recognition site with a neighboring miRNA recognition site within 50 nts, the rescue count was computationally estimated as the number of nucleotides in the miRNA recognition site that PUM binding frees from hybridization with other nucleotides. The distributions of miRNA site rescue counts are shown in histograms for interacting miRNAs (red) and non-interacting miRNAs (gray) in human and mouse. (C, D) For all interacting miRNAs of PUM, the background model (Random) represents the histogram generated when RBP-miRNA paired site sequences were randomly shuffled while preserving mono and di-nucleotide frequency. Standard deviations were estimated from 10 randomizations.

For each RBP, we plotted the histogram of miRNA site rescue counts for RBP sites in close proximity to recognition sites for interacting miRNAs, and non-interacting miRNAs (Figure 5A, B and Figure S13A, B). When we performed this analysis for PUM, interacting miRNAs produced significantly higher rescue counts than non-interacting miRNAs in both human and mouse (Figure 5A, B, Wilcoxon *p*-value<1E-10 for both human and mouse). We also performed a control in which, for interacting miRNAs, the sequence of the miRNA and RBP recognition sites were shuffled while preserving mono and di-nucleotide frequency [66], [67]. For PUM, the proportion of RBP recognition sites with large rescue counts was consistently higher in the true histogram than in the background model, while the proportion of smaller rescue counts was depleted (Figure 5C, D and Figure S14, Wilcoxon *p*-value<1E-10 for both human and mouse). For UAUUUAU, we did not observe any enrichment of high rescue counts for interacting miRNAs compared with controls (Figure S13). In summary, miRNA recognition sites located near a PUM site have a significantly increased frequency of high recognition site rescue by simulated PUM binding than expected by chance.

### miRNAs that interact with PUM have recognition seeds reverse complementary to the PUM recognition motif

**Figure 6:**
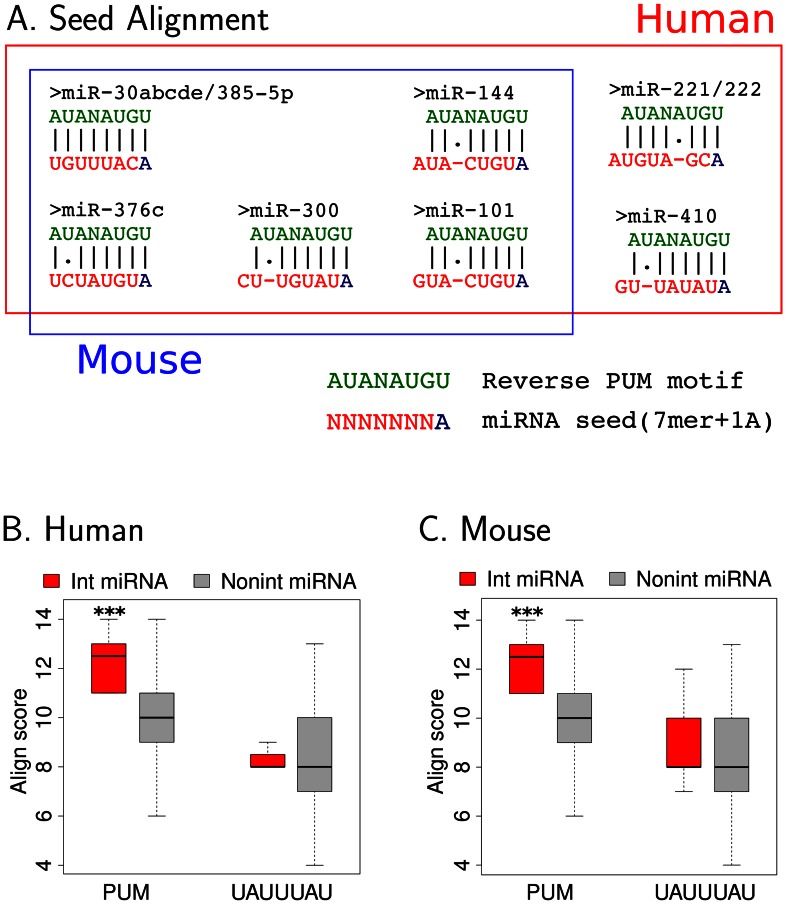
PUM-interacting miRNAs have seed sequence complementarity to the reverse PUM recognition motif. (A) Optimal complementary alignments between miRNA recognition seed sequences and the reverse PUM motif are shown for interacting miRNAs in both human and mouse (Figure 3). Nucleotides 2 to 8 of the miRNA seed site sequence are highlighted in red and the first adenosine position is colored in blue [6]. The reverse PUM recognition motif is colored in green. (B, C) For each miRNA seed, a score was determined based on the extent of complementary base pairing with the reverse RBP recognition motifs [68]. Higher scores indicate better matches with the reverse complementary RBP motif. For each RBP, box-plots of alignment scores are shown for interacting miRNA seeds and non-interacting miRNA seeds. The bottom and top of the box are the 25th and 75th percentiles (the inter-quartile range). Whiskers on the top and bottom represent the maximum and minimum data points within the range represented by 1.5 times the inter-quartile range. For each RBP, asterisks represent comparisons of alignment scores between “Int miRNA” and “Nonint miRNA” by Wilcoxon test. One asterisk indicates *p*<0.05, two asterisks indicate *p*<0.01, and three asterisks indicate *p*<0.001.

We derived a score to measure the ability of miRNA recognition seed sequences to hybridize with the reverse PUM recognition motif, an association that would result in RNA hairpin loop structures in the target mRNA, based on sequence alignment [68] (Figure 6A). A larger score indicates that there is more nucleotide complementarity between the miRNA seed sequence and the reverse of the PUM recognition motif. We found that PUM-interacting miRNAs have significantly higher alignment scores than non-interacting miRNAs in both human and mouse (Figure 6B, C). Thus, if a miRNA co-occurs with PUM recognition sites, it has a higher potential to pair up with the reverse PUM sequence. For UAUUUAU, there was no difference in the alignment scores for interacting versus non-interacting miRNAs (Figure 6B, C).

By comparing real and shuffled PUM motifs, we found that the reverse recognition motifs for PUM tend to have larger alignment scores with interacting miRNA seed sequences than shuffled PUM motifs (Figure S15). Thus, the observed enrichment of higher miRNA rescue counts for PUM is likely to derive from its reverse complementarity with a specific group of miRNA seeds that also have recognition sites preferentially co-localized with PUM recognition sites. For all interacting miRNAs in human or mouse, we diagrammed their 8mer seed (7mer+1A [6]) sequences aligned to the reverse PUM motif (Figure 6A).

## Discussion

### Prevalent models for RBP-miRNA interactions

Some previous studies on RBP-miRNA interactions have experimentally demonstrated specific instances in which RBPs and miRNAs compete with each other, sometimes for the same binding site [20], [24], [25]. In this model, the presence of a RBP recognition site would protect the associated transcript from miRNA-mediated decay and stabilize it. However, this mode of interaction does not seem to be prevalent among the RBP-miRNA interactions we uncovered from our transcriptome-wide analysis as the presence of both a recognition site for a RBP and a miRNA did not result in a global shift toward more stable transcripts using the methodology we employed.

Another model for RBP-miRNA interactions involves RBPs binding closely to miRNA sites and altering the local secondary structure to make miRNA sites more accessible to the RISC complex [29], [63]. When PUM was computationally folded with nearby miRNA recognition sites, the presence of the RBP resulted in increased availability of the miRNA recognition sites. For PUM, the rescue counts were higher for the interacting miRNA sites than for non-interacting miRNAs and background models (Figure 5).

For the PUM recognition site, we are able to develop a computational model to explain the miRNA-specific interactions based on reverse complementarity between the recognition seeds for miRNAs and the PUM recognition motif, which is advantageous for formation of hairpin loops (Figure 6). A previous report described a case study in which miR-221/222 pairs up with the PUM recognition sequence to achieve condition-specific miRNA-mediated decay of the p27^Kip1^, based on PUM expression and its phosphorylation state [29]. Our analysis indicates that the mechanism described for this particular case may also occur for other miRNAs. Further, selective pressure for this mechanism may have shaped the localization pattern of a group of miRNAs by enriching them to be close to PUM recognition sites. Regulation of the levels or activity of RBPs represents a previously unappreciated mechanism for increasing or decreasing the efficiency of many miRNA binding sites simultaneously.

For the ARE element UAUUUAU, we identified a group of miRNAs that are enriched in their co-localization with its recognition sites (Figure 3). These sites may have a function because UAUUUAU motifs are more evolutionarily conserved if they are proximal to an interacting miRNA recognition site (Figure 4C, D) and they did promote more rapid decay, although the effect was not as significant as the effect observed for PUM (Figure 4A, B). However, the presence of UAUUUAU demonstrated no capacity to rescue miRNA binding sites from secondary structure. Our data suggest that UAUUUAU may cooperate with nearby miRNAs to affect transcript decay through a different mechanism, but more studies will be needed to clarify whether there is an effect of proximal UAUUUAU-interacting miRNA sites on transcript decay and its mechanistic basis.

In sum, our results estimated the prevalence of synergistic interactions between PUM and miRNAs. Some previous observations about miRNA targeting, including the efficiency of miRNA recognition sites in 3′UTRs, in AU-rich regions and at the beginning and end of the 3′UTR may be partially explained by synergistic interactions with RBPs [6], [69]. Currently, 829 human proteins are annotated as having RNA binding capacity by Gene Ontology [70] and we have only investigated the small fraction of them for which recognition site information is available. Other RBPs may also mediate the accessibility of miRNA recognition sites. A more comprehensive understanding of the interactions between miRNAs and RBPs could improve our ability to predict their targets and physiological functions, and provide insight into the mechanistic basis for their action.

## Methods

### Multiple genome alignment and 3′UTR annotation

Gene annotations for human, mouse, fly and worm were downloaded from the UCSC genome browser (http://genome.ucsc.edu/). Multiple genome alignments for the human genome (hg19) aligned with 32 placental mammals and mouse genome (mm9) with 29 vertebrates were also downloaded from the UCSC genome browser. 3′UTR regions were extracted for further analysis. Branch lengths for the associated phylogenetic tree were also downloaded.

The 3′UTR was defined as the region between the last stop codon and the 3′ end of the spliced mRNA. In some unusual cases, 3′UTRs are formed by the union of several distinct exons and cannot be mapped to a single continuous region on the genome assembly. For ease of analysis, we considered the last spliced exon, excluding any overlap with the protein-coding region, to be the 3′UTR. We also required that each 3′UTR was longer than 10 nts. In total, we analyzed 18,854 human 3′UTRs with average length 1,292.3 +/− 1,480.3 as standard deviation. Among these 3′UTRs, 17,766 were initiated at the stop codon and had no overlap with any coding region, and thus 94.2% of the 3′UTR annotations are complete.

### RBP and miRNA recognition motif selection

All RBP binding motifs were first converted to consensus sequences. We expressed the consensus sequences as regular expressions, and used the regular expressions to search for recognition sites within the genomic sequence. We searched the 3′UTRs of the transcribed strands in multiple genome alignments for RBP recognition motifs in all aligned sequences. For each motif hit in the reference genome, we determined whether the same recognition motif was present within 10 nts in either direction in each of the other genomes. Based on the presence or absence of the motif within the investigated genomes, we calculated the branch length score (BLS) by defining the minimum phylogenetic sub-tree that includes all conserved instances of the motif. The BLS is the branch length of this sub-tree as a fraction of the entire tree. Using this method, the BLS of individual motif hits can be inflated by a single hit to a distant species. To avoid this, we assigned no score to the most distant hit if there was a gap to that species that included more than one-third of the number of aligned genomes, and if the evolutionary distance from the most distant genome to the reference genome was more than twice as large as the distance to the second-most distant genome.

In order to assess the extent of conservation for each RBP motif, we generated 200 shuffled motifs by randomly swapping the nucleotides within the recognition motif. To remove redundant shuffled motifs, we profiled the similarity of each pair of motifs using Tomtom to generate *p*-values among the canonical and shuffled recognition motifs [71]. We ranked the *p*-values and determined the 10% threshold for the pairs with the most similar *p*-values. We eliminated shuffled motifs if they fell within this range and thus were considered too similar to any previously generated shuffled motif or the canonical motif. From the remaining motifs, we selected ten or the maximum possible number of shuffled motifs. When possible, we selected shuffled motifs that had a similar number of hits (±20%) to the canonical motif from the reference genome. Through these criteria, we largely corrected for differences in the frequencies of di and tri-nucleotides [72]. For certain recognition motifs, nearly all shuffled motifs were associated with significantly fewer hits than the canonical motif. In this case, we selected 10 or the maximum possible number of arbitrary shuffled motifs as controls. For some RBP motifs with low complexity, for instance, sequences that were represented by a string of U's, we could not create three distinct shuffled motifs. These motifs were eliminated from further analysis.

We compared the conservation BLS scores for the canonical motif and each shuffled motif within the genome. We then determined the number of occurrences of hits to the genome for the canonical motifs and the average among shuffled motifs for 100 different BLS thresholds from 0 to 1 with increments of 0.01. For each BLS threshold, we determined the precision as 1 – (the average number of matches of shuffled motifs)/(the number of matches of the canonical motif). We selected for further analysis RBPs for which the recognition motif contained more than 10 motif hits above a precision threshold of 0.6.

Mature human, mouse, fly and worm miRNA annotations were downloaded from Targetscan (http://targetscan.org). Two types of miRNA seeds were used: miRNA nucleotides 2–8 (m8) and nucleotides 2–7 with an adenosine opposite miRNA position 1 (1A) [6]. miRNA recognition seed motifs were defined as the complements of the miRNA seed and only conserved miRNA families were considered in further analyses.

### RBP-miRNA motif site interaction

For each pair of miRNA and RBP, we defined the position of the RBP recognition motifs and identified the locations of the neighboring miRNA recognition sites in either direction. We generated histograms to depict the frequency with which the closest miRNA recognition motifs were present at ten different 50 nt windows 5′ and 3′ of the RBP motif. To generate a background model, we shuffled the identities of the miRNAs within each chromosome while keeping their positions intact. By shuffling the miRNA identities, we specifically tested the importance of co-localization with that particular miRNA. This approach eliminates any bias introduced by the fact that miRNA binding sites tend to be present together. Ten thousand shuffles were generated.

For each RBP, in each 50-nt-window, we compared the number of miRNA recognition sites for the real distribution versus the number derived from shuffled distributions. For each miRNA seed, the empirical *p*-value was calculated as the proportion of times that the number of miRNA sites was equal to or larger than the real number of miRNA sites when 10,000 shuffles were performed. We then applied the Benjamini-Hochberg procedure on the *p*-values, and selected interacting miRNAs for each RBP with a FDR< = 0.05 [57]. Since for each miRNA there are two possible types of miRNA recognition seeds (1A and m8) [6], we required both of them to pass the FDR threshold of 0.05 to be included as an interacting miRNA.

Both RBPs and miRNAs tend to have recognition sites located at the beginning or end of 3′UTRs (Figure 2) [48], [49]. The miRNAs are more effective when localized in AU-rich regions [6]–[8]. Further, several of the RBP recognition motifs investigated have high AU content, with the most extreme instance being UAUUUAU. Thus, it is possible that the RBP-miRNA site colocalization we observed is a reflection of the similar positional preference of RBPs and miRNAs or their similarity with respect to the AU-richness of both types of motifs. In order to control for positional preference and AU content, we derived additional miRNA site identity shuffling procedures. To control for positional preference, all 3′UTRs were equally divided into 10 deciles and miRNA recognition sites were grouped according to the 3′UTR decile to which they belong. Then the identities of the miRNAs were shuffled among each 3′UTR decile group; in this way, miRNA sites located at the very end (or beginning) of 3′UTRs were swapped exclusively with other miRNA sites located at the very end (or beginning) of 3′UTRs.

To control for AU content, miRNA recognition seeds of 1A and m8 were classified into 3 groups based on the number of nucleotides that are an A or U out of the seven base pairs in the seed sequence. Category 1 contained miRNAs with a high (6 or 7) number of A/U nucleotides; category 2 contained miRNAs with a medium (3–5) number of A/U nucleotides; and category 3 contained miRNAs with a low (0–2) number of A/U nucleotides. The identities of miRNAs were shuffled with other miRNAs within the same category. In this way, AU-rich miRNA recognition sites were swapped exclusively with other AU rich miRNA sites. Empirical *p*-values and Benjamini-Hochberg correction were performed as described above.

We only accepted miRNAs identified by the intersection of all three methods as interacting miRNAs for each RBP in each window. We found the window of 50 nts closest to the RBP site contained the largest number of interacting miRNAs, while windows that were more distant contained fewer or none (Table S1). To compile our final set of interacting miRNAs for each RBP, we only considered the miRNAs identified in the first 50 nt window. For each 50 nt window, an enrichment ratio was defined and visualized (Figure 3A) as the minimum ratio of (number of miRNA sites)/(expected number) among the three different shuffle methods.

Detailed statistics for all possible pairs of RBP and miRNA recognition motifs are available on the webpage http://cat.princeton.edu/miRNA_RBP/.

### Test for the effect of AU content on RBP-miRNA interaction

When defining interacting miRNAs for each RBP, we explicitly accounted for the AU content of miRNAs by only shuffling across miRNAs with similar recognition seed AU content. In order to test the impact of the AU content of the RBP motifs, we generated window plots comparing the distribution of true RBP motifs to the distribution of shuffled RBP motifs (generated in the RBP recognition motif selection section). We created ten 50 nt windows upstream and downstream of each RBP motif and shuffled RBP motifs and counted the number of real miRNA recognition sites. For each window, the enrichment ratio was defined as ([Number of pairs for real RBP site and real miRNA site]/[Number of pairs for shuffled RBP site and real miRNA site]) normalized by an overall ratio of ([Number of pairs for real RBP site and real miRNA site across all windows]/[Number of pairs for shuffled RBP site and real miRNA site across all windows]). For each RBP and its interacting miRNAs, enrichment ratios were visualized with heatmaps (Figure S8).

### Cell-type specific expression profiles of miRNAs

For the cell lines included in our analysis, we identified companion, published small RNA sequencing experiments. For mRNA half-life measurements in human B cells (BL41) [58], miRNA sequencing reads were analyzed from the dataset generated by Landgraf and colleagues [60]. For mRNA half-life measurements in mouse fibroblasts (NIH-3T3) [58], miRNA reads were analyzed from the dataset generated by Zhu and colleagues [61]. For mRNA decay rate measurements in human HepG2 [59], miRNA reads were analyzed from the dataset generated by the ENCODE project [62]. For Par-CLIP datasets of PUM2 and AGO binding, small RNA sequencing data was analyzed from the dataset generated by Hafner and colleagues [37].

For each sequencing experiment, all conserved miRNAs annotated in TargetScan were ranked by the number of their mapped sequence reads. The most highly expressed 25% of the miRNAs were defined as expressed and the rest were defined as non-expressed.

### Effects of RBPs on miRNA site accessibility

To test whether the binding of RBPs makes miRNA recognition sites more accessible, we analyzed sequences that contain RBP and miRNA recognition sites within 50 nts of each other, and included an extra 5 nts upstream of the 5′ most site and 5 nts downstream of the 3′ most site. We computationally folded these sequences using RNAfold [64] and determined the count C1 as the number of base pairs within the miRNA recognition seed site that are paired. Then, we converted all of the nucleotides within the RBP recognition site and one flanking nucleotide on each side to an ‘N’ to mask them from pairing [63]–[65], and reran RNAfold to determine the number of base pairs within the miRNA site that were paired as count C2. For RNAfold predicted structure with folding energy larger than −1 kcal/mol, we considered this structure to be totally open and ignored any base pairing predicted. Finally, we calculated the rescue count C = C1–C2.

In order to generate a background distribution of rescue counts, for each pair of neighboring miRNA and RBP sites localized within 50 nts, we randomized the sequence itself, but preserved the relative positions of the miRNA and RBP recognition sites. For this randomization, we preserved the mono and di-nucleotide frequency [66], as RNA folding energy is known to depend on di-nucleotide base stacking energies, and certain known RNA structures, such as tRNAs, have indistinguishable folding energy from di-nucleotide-preserving shuffles [67]. After randomizing the sequence for all miRNA-RBP neighboring sites, we repeated the rescue count calculation again. Ten randomizations were generated to estimate the average and standard deviation of the rescue counts in the background model.

To score the ability of miRNA recognition seed sequences to hybridize with the reverse RBP recognition motif, we used a simple scoring scheme in which A-U, G-C and G-U base pair matches were scored as 2, mismatches were scored as 0, and insertions and deletions were penalized with −1. The Smith-Waterman algorithm was then applied to find the best alignment [68].

## Supporting Information

Figure S1 **RBP recognition motifs.** The positional weight matrix logos are plotted for the 15 RBP motifs that were evaluated.
(PDF)

Figure S2 **RBP recognition motif selection.** The number of instances of each RBP was plotted for different Branch Length Scores (BLS) as in Figure 1. These values are plotted using the y-axis on the left. True motifs are indicated in green and shuffled motifs are indicated in gray. Precision is shown in red and plotted according to the y-axis on the right. (A) UAUUUAU. (B) U1A. (C) Nova. (D) U2B.
(PDF)

Figure S3 **AU-content is high at the end of 3′UTRs.** Each 3′UTR longer than 500 nts was equally divided into ten deciles. For each decile, AU-content was calculated and box-plots across all genes are shown. (A) Human. (B) Mouse. (C) Fruit fly. (D) Worm.
(PDF)

Figure S4 **RBP localization compared with shuffled control motifs.** Each 3′UTR longer than 2000 nts was equally divided into ten bins, numbered from 0.0 to 0.9. The percentage of RBP recognition sites in each bin was compared with its shuffled control motifs, which have the same AU-content. For each 3′UTR bin, asterisks represent comparisons of percentages between real and random motifs using the binomial test with a Bonferroni correction for 10 tests. One asterisk indicates *p*<0.05, two asterisks indicate *p*<0.01, and three asterisks indicate *p*<0.001. (A) PUM localization in human. (B) UAUUUAU localization in human. (C) PUM localization in mouse. (D) UAUUUAU localization in mouse.
(PDF)

Figure S5 **Localization of RBP motifs in 3′UTRs across four organisms.** For each organism, 3′UTRs with length longer than 500 nts, but shorter than 2000 nts, were considered. Each 3′UTR was equally divided into 10 bins, numbered from 0.0 to 0.9. The percentage of RBP recognition sites in each bin was plotted. (A) PUM localization pattern. (B) UAUUUAU localization pattern.
(PDF)

Figure S6 **Percentage of miRNA recognition sites within 50 nts from RBP sites.** For each RBP, the percentage of miRNA recognition sites within 50 nts of an RBP recognition site was determined for the set of all interacting miRNAs (Figure 3) and for the set of all non-interacting miRNAs. All values are shown with box-plots. For each RBP, asterisks represent comparisons of percentages between “Int miRNA” and “Nonint miRNA” determined by Wilcoxon tests. One asterisk indicates *p*<0.05, two asterisks indicate *p*<0.01, and three asterisks indicate *p*<0.001. Two separate data plots are shown for (A) Human and (B) Mouse.
(PDF)

Figure S7 **PUM co-localizes with its interacting miRNAs in the Par-CLIP region.** The recognition sites of PUM and miRNAs were restricted to those experimentally identified by Par-CLIP analysis of PUM2 and AGO binding in the HEK293T cell line [37]. The number of neighboring PUM and miRNA recognition sites within 50 nts was counted. To determine the number of neighboring recognition sites expected by chance, the labels of all miRNA recognition sites were shuffled across chromosomes and the number of neighboring PUM and miRNA sites within the Par-CLIP region was counted again. For each miRNA, the enrichment ratio was calculated as (#neighboring sites)/(#expected sites). (A) Enrichment ratios for PUM-interacting miRNAs (Figure 3A) and non-interacting miRNAs are shown with box-plots. Asterisks represent comparisons of enrichment ratios between the two groups determined by Wilcoxon tests. One asterisk indicates *p*<0.05, two asterisks indicate *p*<0.01, and three asterisks indicate *p*<0.001. (B) The PUM-interacting miRNAs were classified as expressed if they were among the 25% of the most frequently sequenced small RNAs in HEK293T cells [37] (miR-30abcde/384-5p, miR-101 and miR-221/222). The rest of the PUM-interacting miRNAs were classified as non-expressed (miR-144, miR-300, miR-376c, miR-410). Enrichment ratios between the two groups were visualized and compared in the same way as described in (A).
(PDF)

Figure S8 **miRNA-RBP colocalization is not simply a consequence of AU-content.** For RBPs and their interacting miRNAs (Figure 3), we considered each of ten 50-nt windows upstream and ten 50-nt windows downstream of a RBP binding motif. We determined the enrichment ratio of the number of miRNA recognition sites located in that window compared to the number of miRNA sites localized to shuffled RBP motifs with the same nucleotide content, normalized by their overall numbers across all 50-nt windows (Methods). The enrichment ratio in each window is shown in heatmap format. (A) Human enrichment heatmap. (B) Mouse enrichment heatmap.
(PDF)

Figure S9 **The presence of PUM and UAUUUAU results in faster transcript decay.** Decay rates are shown in box-plots for transcripts with recognition sites for the designated RBP or shuffled RBP motifs. The Wilcoxon-test with a Bonferroni correction was applied to measure the difference between real RBP sites (Real) and shuffled RBP controls (Random). For each RBP, an asterisk designates a significant difference between transcripts with RBP recognition sites and transcripts with shuffled RBP motif sites. One asterisk indicates *p*<0.05, two asterisks indicate *p*<0.01, and three asterisks indicate *p*<0.001. (A, B) Half-lives are based on the published dataset [58]. (C) Decay rates are based on the published dataset [59].
(PDF)

Figure S10 **Pumilio recognition sites promote decay more effectively and are better conserved when present with interacting miRNAs.** For each RBP, miRNAs were classified into four groups as described for Figure 4. (A) Decay rates were plotted based on dataset [59] as described in Figure 4A, B. (B) Conservation BLS scores were calculated based on ten primate species alignment, and plotted as described in Figure 4C, D.
(PDF)

Figure S11 **Expressed miRNAs promote decay more effectively than non-expressed miRNAs.** For each of the cell lines used in half-life or decay rate datasets, companion small RNA sequencing datasets were identified from the literature. In each dataset, the reads of miRNAs were ranked and the most frequently expressed 25% of small RNA reads was established as a threshold for classifying interacting miRNAs for each RBP as “Expressed” or “Non-expressed”. Transcripts with proximal miRNA and RBP sites were compared with respect to their half-lives or decay rates. Asterisks represent comparisons of half-lives between two groups determined by Wilcoxon tests. One asterisk indicates *p*<0.05, two asterisks indicate *p*<0.01, and three asterisks indicate *p*<0.001. (A) For mRNA half-lives measured in Human B cells (BL41) [58], miRNA sequencing reads were derived from a dataset generated by Landgraf and colleagues [60]. (B) For mRNA half-lives measured in mouse fibroblasts (NIH-3T3) [58], miRNA sequencing reads were derived from dataset generated by Zhu and colleagues [61]. (C) For mRNA decay rates measured in human HepG2 [59], miRNA sequencing reads were derived from dataset generated by the ENCODE project [62].
(PDF)

Figure S12 **More rapid mRNA decay in transcripts in which Pumilio and interacting miRNA recognition sites colocalize is not only a consequence of AU-content.** For each RBP or miRNA recognition motif, the shuffled RBP or miRNA motifs were used as controls for AU content. For each group of transcripts, boxplots of half-lives or decay rates were plotted as described for Figure 4A, B. Group “Real” contained transcripts with at least one RBP recognition site and a recognition site for one of the RBP's interacting miRNA within 50 nts. Group “miR control” contained transcripts with at least one RBP recognition site and a recognition site of shuffled interacting miRNA motif within 50 nts. Group “RBP control” contained transcripts with at least one recognition site of a shuffled RBP motif and an associated interacting miRNA recognition site within 50 nts. Asterisks represent comparisons of half-lives between the groups “Real” and “miR control” determined by Wilcoxon tests. One asterisk indicates *p*<0.05, two asterisks indicate *p*<0.01, and three asterisks indicate *p*<0.001. (A, B) Half-life data from Friedel and colleagues [58], (C) Decay rate data from Yang and colleagues [59].
(PDF)

Figure S13 **Histograms of rescue counts for miRNA recognition sites upon UAUUUAU binding.** Histograms of site rescue were calculated for both UAUUUAU-interacting miRNAs and non-interacting miRNAs as described in Figure 5. (A, B) Comparison of histograms between interacting miRNAs and non-interacting miRNAs are shown separately for human and mouse. (C, D) Comparison of histograms between real rescue counts and random rescue counts determined based on a background model are shown for human and mouse.
(PDF)

Figure S14 **PUM rescues nucleotides in neighboring interacting miRNA recognition sites.** The histogram of miRNA seed rescue was compared with rescue values calculated with the background model of Figure 5C, D. Data for each of the seven interacting miRNAs of PUM are shown separately for the human genome. (A) miR-30abcde/384-5p. (B) miR-144. (C) miR-300. (D) miR-101. (E) miR-376c. (F) miR-221/222. (G) miR-410.
(PDF)

Figure S15 **Histograms of alignment scores between miRNA seeds and Pumilio recognition motifs.** For all interacting miRNAs of PUM, their seed alignment scores with the reverse PUM motif were determined for real PUM motif and shuffled PUM motifs. Histograms for all shuffled PUM motifs were merged into average values and standard deviations, and are shown for PUM interacting miRNAs identified in (A) Human and (B) Mouse.
(PDF)

Table S1 **RBP and miRNA recognition motifs colocalize.** For each RBP, the number of predicted interacting miRNAs is shown for each of ten 50-nt-windows from the RBP motif.
(PDF)

Table S2 **AU composition of RBP recognition motifs and interacting miRNA recognition seeds.** The fraction of nucleotides that are AU was calculated for each RBP recognition motif and the recognition seeds of miRNAs that interact with each RBP. The average value is shown. The average AU fraction for all miRNA seeds is 52.7%.
(PDF)

Table S3 **miRNA sites are enriched around RBP sites in human and mouse 3′UTRs.** For each organism, the ratio of miRNA recognition site density 50 nts upstream or downstream of the RBP recognition sites to miRNA site density across all 3′UTRs is reported for PUM and UAUUUAU.
(PDF)

Table S4 **Expressed RBP-interacting miRNAs and their effects on mRNA decay.** For each of the mRNA decay datasets, we considered each RBP and its expressed interacting miRNAs (corresponding to Figure 4A, B). Proximal and distant pairs of recognition sites were defined as in Figure 4. The median mRNA half-lives or decay rates were shown for each combination. *P*-values were calculated from Wilcoxon rank test as a measure of the difference between proximal and distant group. (A) Results from mRNA half-life datasets of human B cells [58]. (B) Results from mRNA half-life datasets of mouse fibroblasts [58]. (C) Results from mRNA decay rate dataset of HepG2 cell line [59].
(PDF)

Table S5 **RBP recognition sites are more conserved when present with interacting miRNAs.** For each RBP and its interacting miRNAs, their neighbor recognition sites were classified into either the proximal or distant groups as described for Figure 4. The median conservation BLS scores are shown for each combination and *p*-values are calculated based on Wilcoxon rank tests as a measure of the difference between the two groups. (A) Human-interacting miRNAs are shown. (B) Mouse-interacting miRNAs are shown.
(PDF)

Table S6 **GO enrichments for PUM and its interacting miRNAs.** Transcripts were classified into categories as in Figure 4. The GO biological process annotations were compared between group “Int-proximal” (transcripts with at least one RBP site and its interacting miRNA recognition site within 50 nts) and group “Int-distant” (transcripts with both RBP sites and its interacting miRNA recognition sites, but no pair of recognition sites is within 50 nts). Hypergeometric enrichment was used to calculate *p*-values. We then applied the Benjamini-Hochberg procedure on the *p*-values, and selected enriched GO terms with FDR< = 0.05. The number of annotated genes and hypergeometric *p*-values are shown for each significant GO term.
(PDF)
