# Supplementary figures and images for: Computational Assessment of the Cooperativity between RNA Binding Proteins and MicroRNAs in Transcript Decay
Source: PLoS Comput Biol. 2013 May 30;9(5):e1003075. doi: 10.1371/journal.pcbi.1003075 (PMC3667768; doi:10.1371/journal.pcbi.1003075)

Supplementary Figure S1

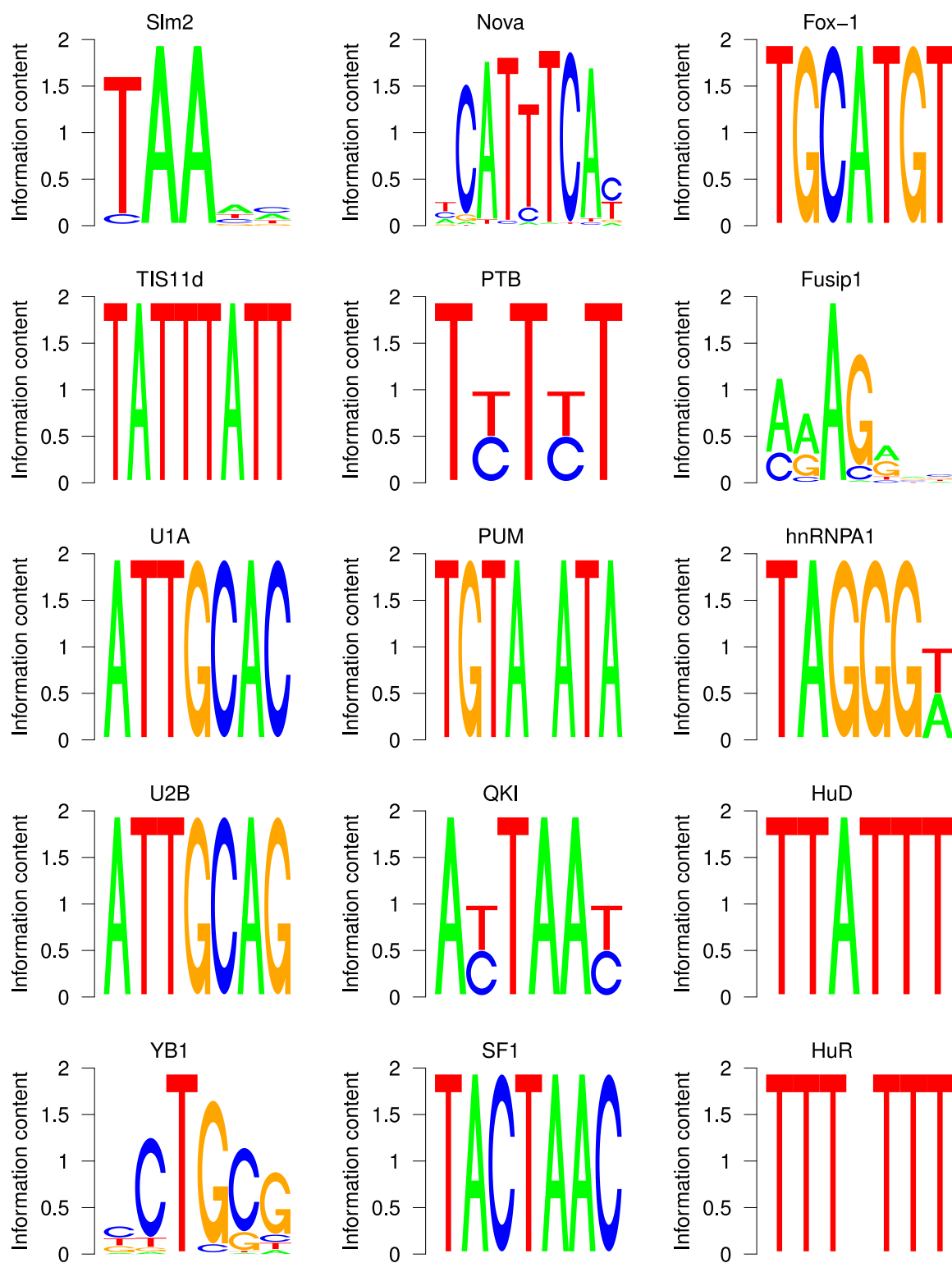

Supplement: Figure S1 — RBP recognition motifs. The positional weight matrix logos are plotted for the 15 RBP motifs that were evaluated. (PDF) [file pcbi.1003075.s001.pdf]

Supplementary Figure S2

A. UAUUUUAU

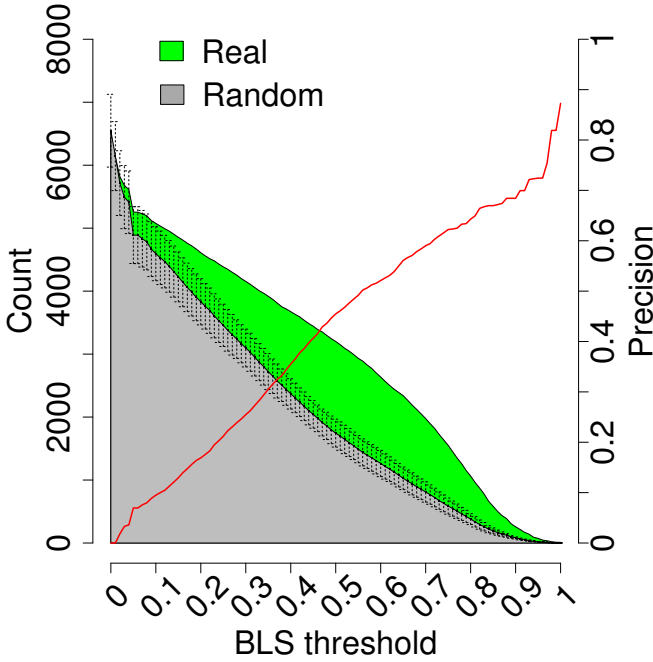

B. U1A

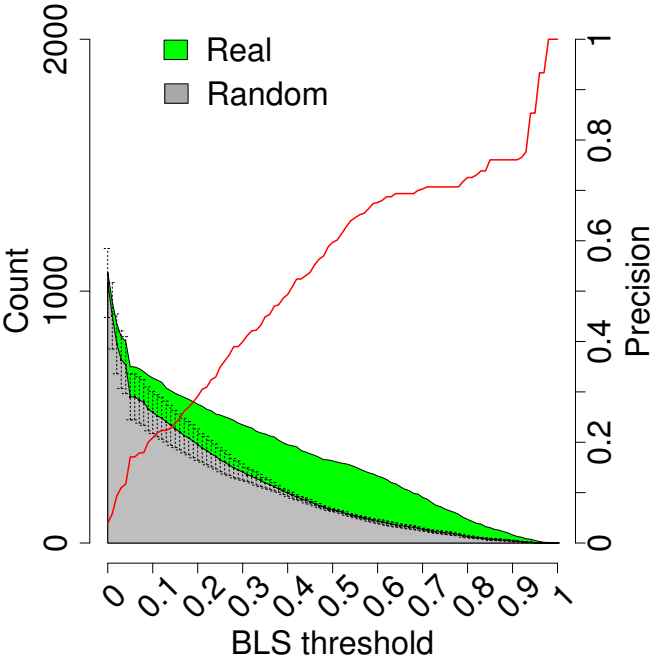

C. Nova

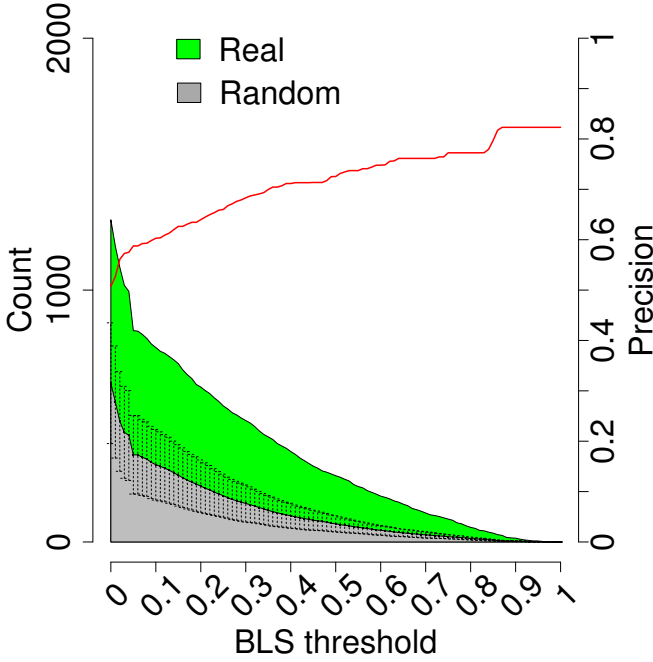

D. U2B

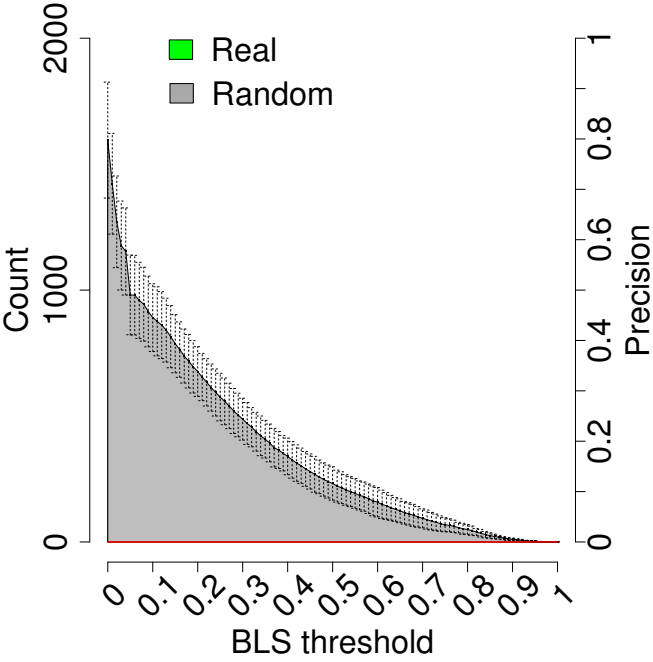

Supplement: Figure S2 — RBP recognition motif selection. The number of instances of each RBP was plotted for different Branch Length Scores (BLS) as in Figure 1. These values are plotted using the y-axis on the left. True motifs are indicated in green and shuffled motifs are indicated in gray. Precision is shown in red and plotted according to the y-axis on the right. (A) UAUUUAU. (B) U1A. (C) Nova. (D) U2B. (PDF) [file pcbi.1003075.s002.pdf]

Supplementary Figure S3

A. *H.sapiens*

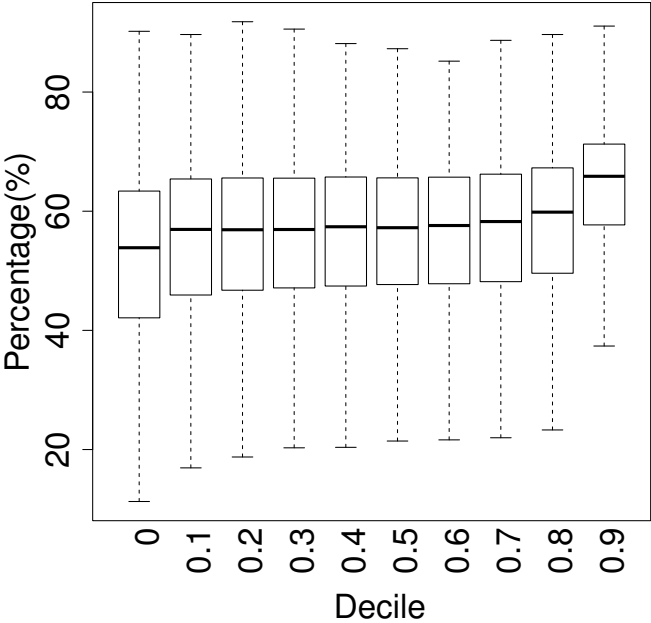

B. *M.musculus*

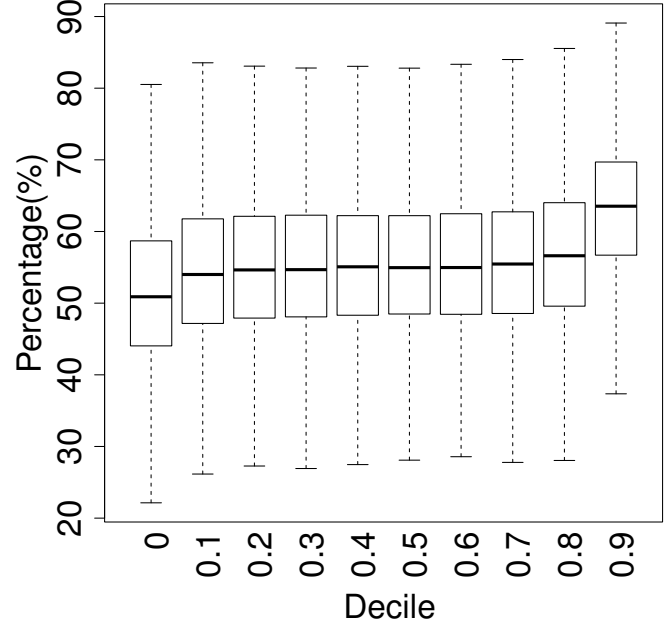

C. *D.melanogaster*

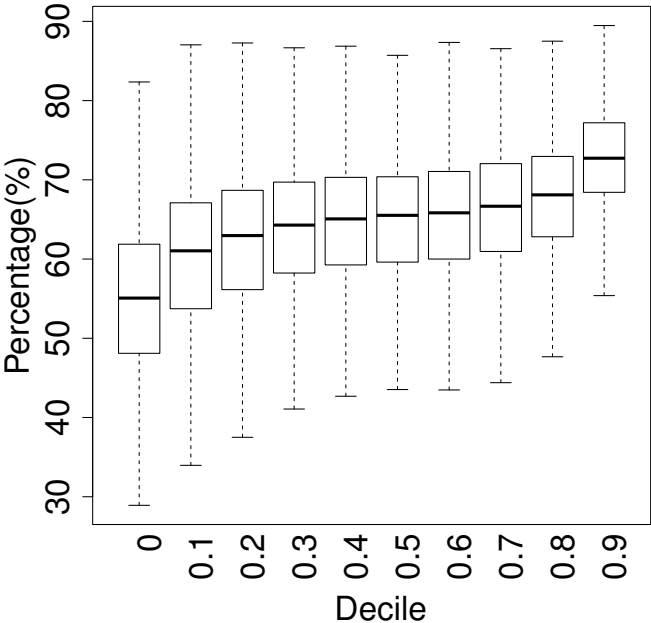

D. *C.elegans*

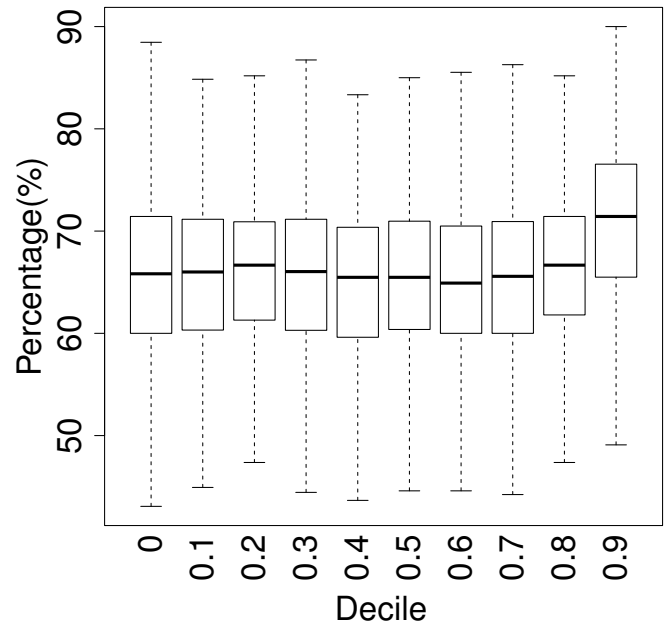

Supplement: Figure S3 — AU-content is high at the end of 3′UTRs. Each 3′UTR longer than 500 nts was equally divided into ten deciles. For each decile, AU-content was calculated and box-plots across all genes are shown. (A) Human. (B) Mouse. (C) Fruit fly. (D) Worm. (PDF) [file pcbi.1003075.s003.pdf]

Supplementary Figure S4

A. Human. PUM

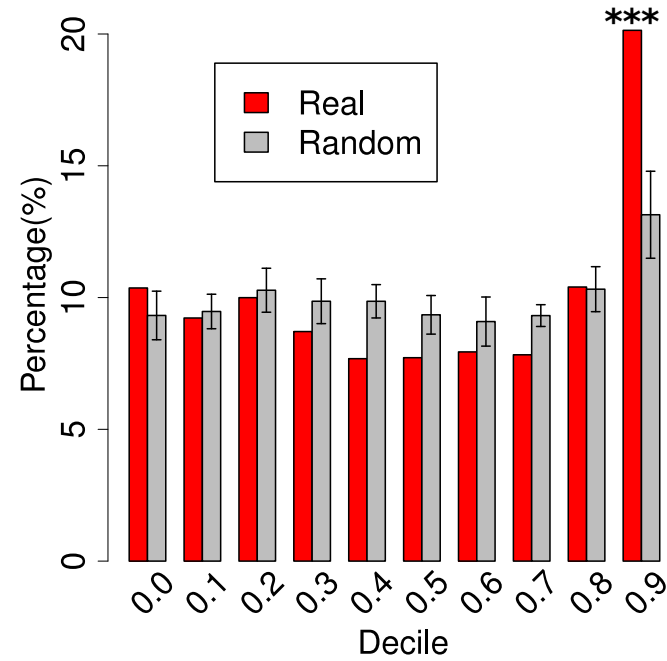

B. Human. UAUUUUAU

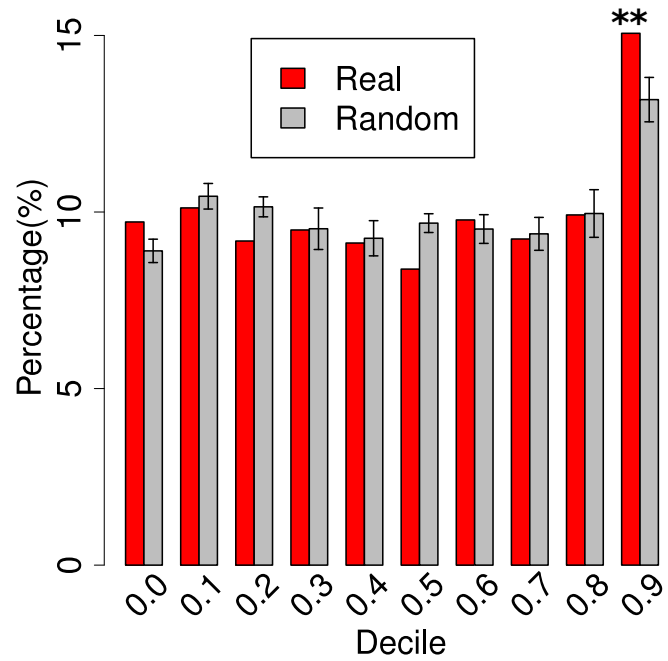

C. Mouse. PUM

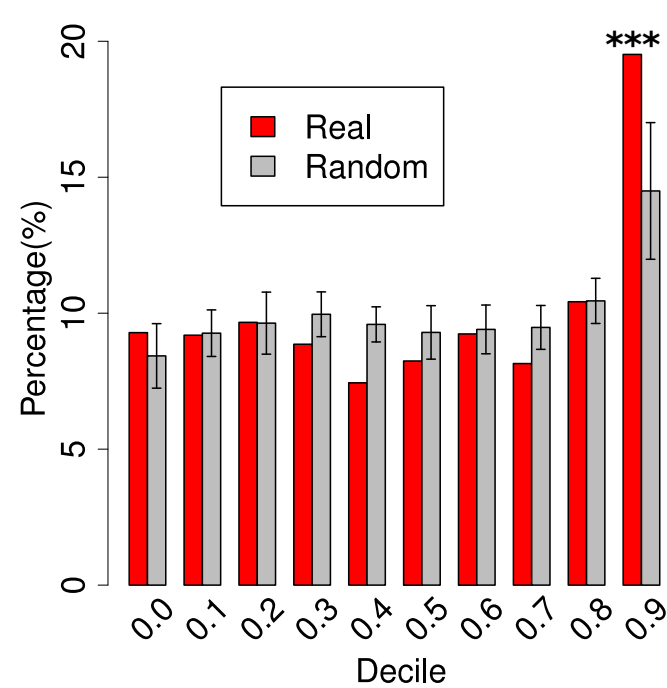

D. Mouse. UAUUUUAU

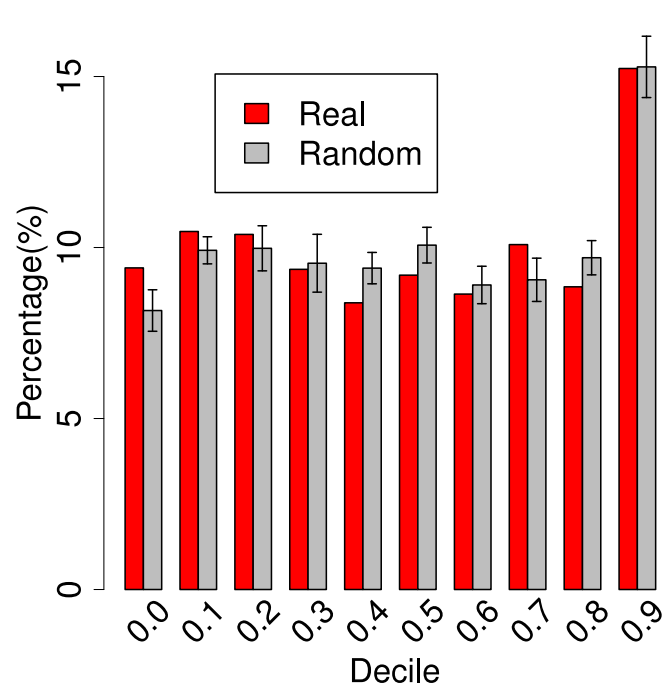

Supplement: Figure S4 — RBP localization compared with shuffled control motifs. Each 3′UTR longer than 2000 nts was equally divided into ten bins, numbered from 0.0 to 0.9. The percentage of RBP recognition sites in each bin was compared with its shuffled control motifs, which have the same AU-content. For each 3′UTR bin, asterisks represent comparisons of percentages between real and random motifs using the binomial test with a Bonferroni correction for 10 tests. One asterisk indicates p<0.05, two asterisks indicate p<0.01, and three asterisks indicate p<0.001. (A) PUM localization in human. (B) UAUUUAU localization in human. (C) PUM localization in mouse. (D) UAUUUAU localization in mouse. (PDF) [file pcbi.1003075.s004.pdf]

Supplementary Figure S5

A. PUM

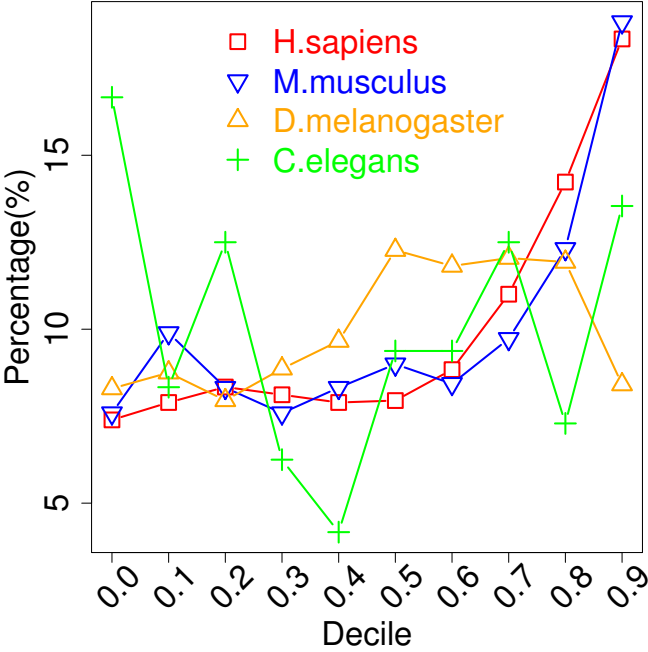

B. UAUUUUAU

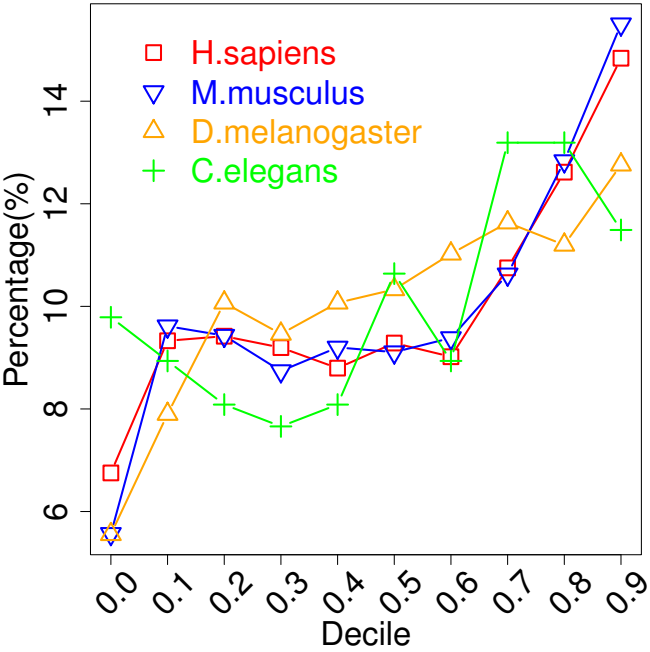

Supplement: Figure S5 — Localization of RBP motifs in 3′UTRs across four organisms. For each organism, 3′UTRs with length longer than 500 nts, but shorter than 2000 nts, were considered. Each 3′UTR was equally divided into 10 bins, numbered from 0.0 to 0.9. The percentage of RBP recognition sites in each bin was plotted. (A) PUM localization pattern. (B) UAUUUAU localization pattern. (PDF) [file pcbi.1003075.s005.pdf]

Supplementary Figure S6

A. Human

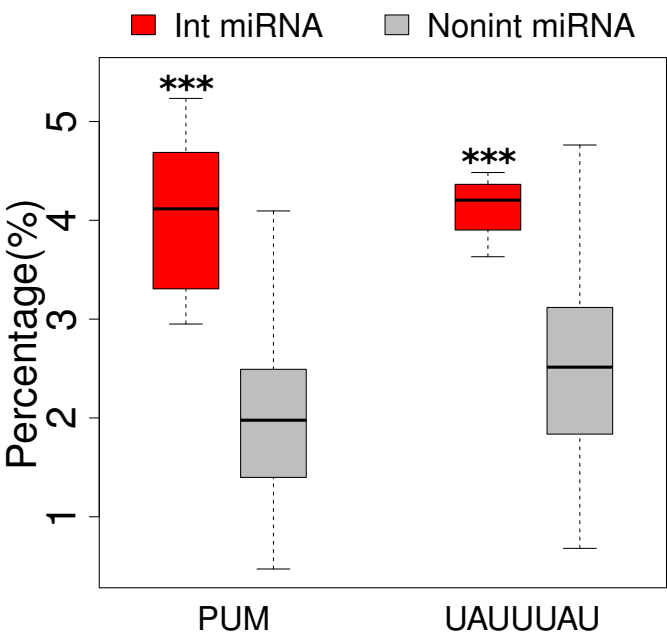

B. Mouse

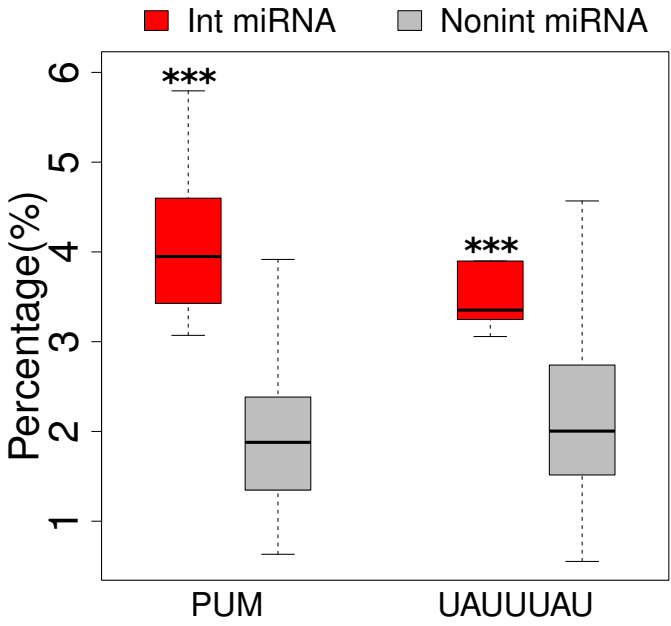

Supplement: Figure S6 — Percentage of miRNA recognition sites within 50 nts from RBP sites. For each RBP, the percentage of miRNA recognition sites within 50 nts of an RBP recognition site was determined for the set of all interacting miRNAs (Figure 3) and for the set of all non-interacting miRNAs. All values are shown with box-plots. For each RBP, asterisks represent comparisons of percentages between “Int miRNA” and “Nonint miRNA” determined by Wilcoxon tests. One asterisk indicates p<0.05, two asterisks indicate p<0.01, and three asterisks indicate p<0.001. Two separate data plots are shown for (A) Human and (B) Mouse. (PDF) [file pcbi.1003075.s006.pdf]

Supplementary Figure S7

A.

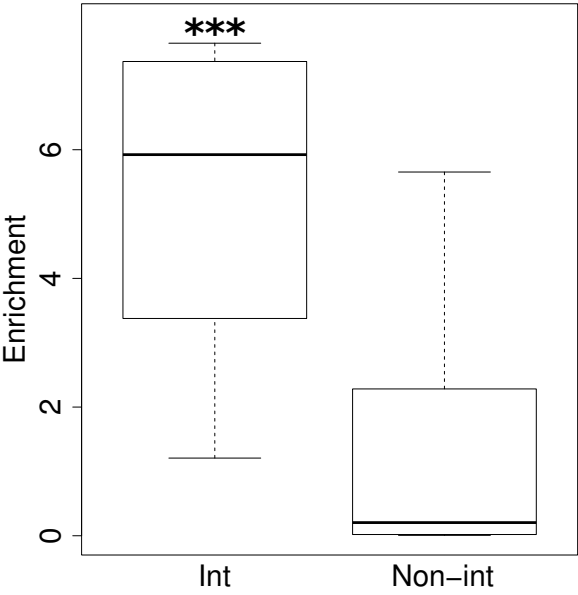

B.

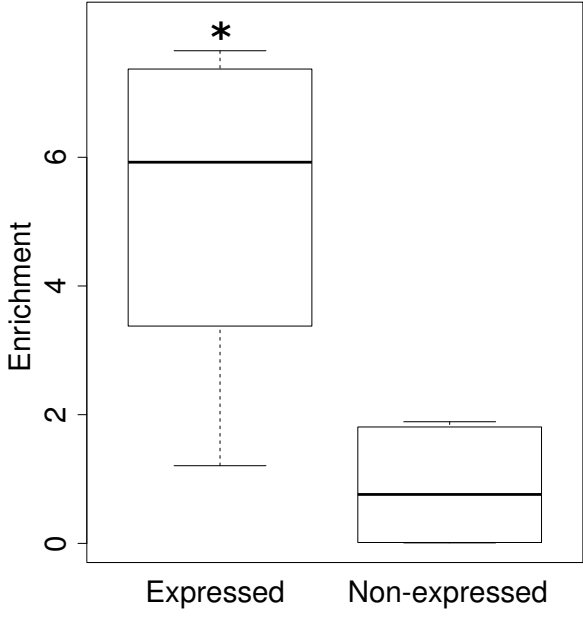

Supplement: Figure S7 — PUM co-localizes with its interacting miRNAs in the Par-CLIP region. The recognition sites of PUM and miRNAs were restricted to those experimentally identified by Par-CLIP analysis of PUM2 and AGO binding in the HEK293T cell line [37]. The number of neighboring PUM and miRNA recognition sites within 50 nts was counted. To determine the number of neighboring recognition sites expected by chance, the labels of all miRNA recognition sites were shuffled across chromosomes and the number of neighboring PUM and miRNA sites within the Par-CLIP region was counted again. For each miRNA, the enrichment ratio was calculated as (#neighboring sites)/(#expected sites). (A) Enrichment ratios for PUM-interacting miRNAs (Figure 3A) and non-interacting miRNAs are shown with box-plots. Asterisks represent comparisons of enrichment ratios between the two groups determined by Wilcoxon tests. One asterisk indicates p<0.05, two asterisks indicate p<0.01, and three asterisks indicate p<0.001. (B) The PUM-interacting miRNAs were classified as expressed if they were among the 25% of the most frequently sequenced small RNAs in HEK293T cells [37] (miR-30abcde/384-5p, miR-101 and miR-221/222). The rest of the PUM-interacting miRNAs were classified as non-expressed (miR-144, miR-300, miR-376c, miR-410). Enrichment ratios between the two groups were visualized and compared in the same way as described in (A). (PDF) [file pcbi.1003075.s007.pdf]

Supplementary Figure S8

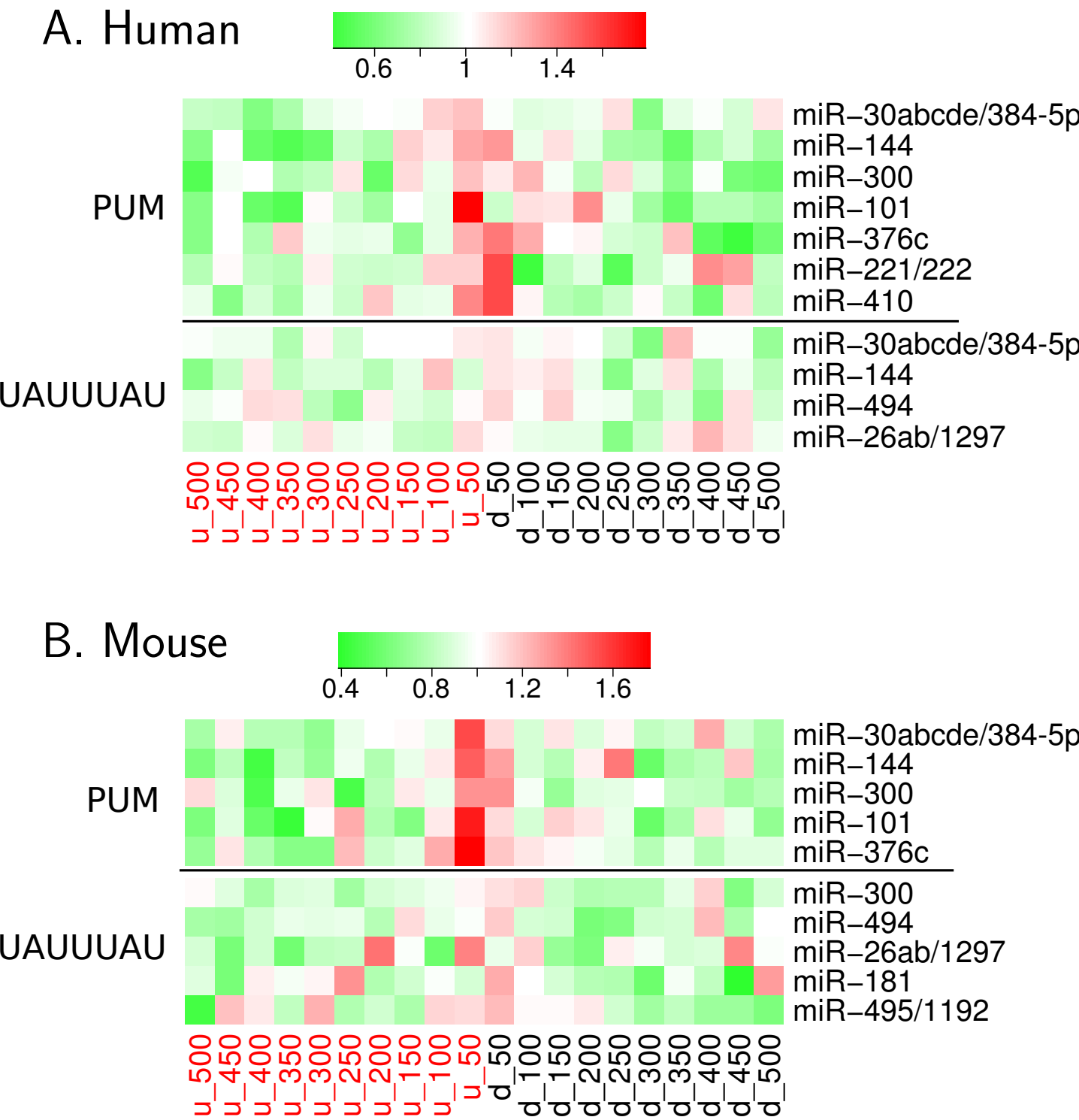

Supplement: Figure S8 — miRNA-RBP colocalization is not simply a consequence of AU-content. For RBPs and their interacting miRNAs (Figure 3), we considered each of ten 50-nt windows upstream and ten 50-nt windows downstream of a RBP binding motif. We determined the enrichment ratio of the number of miRNA recognition sites located in that window compared to the number of miRNA sites localized to shuffled RBP motifs with the same nucleotide content, normalized by their overall numbers across all 50-nt windows (Methods). The enrichment ratio in each window is shown in heatmap format. (A) Human enrichment heatmap. (B) Mouse enrichment heatmap. (PDF) [file pcbi.1003075.s008.pdf]

Supplementary Figure S9

A. Human

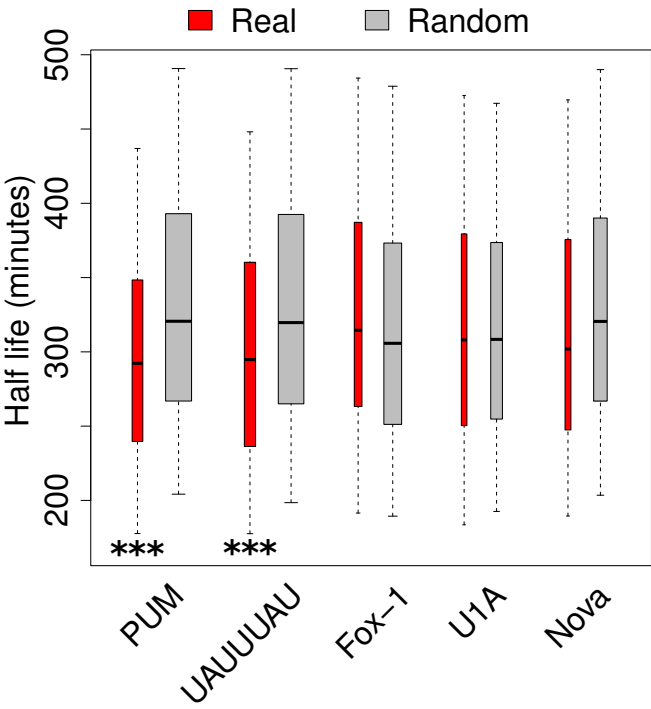

B. Mouse

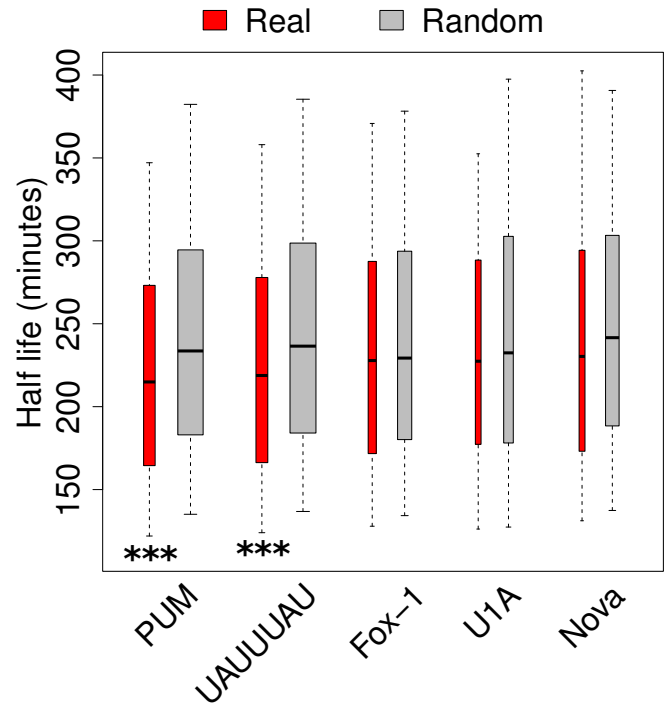

C. Human

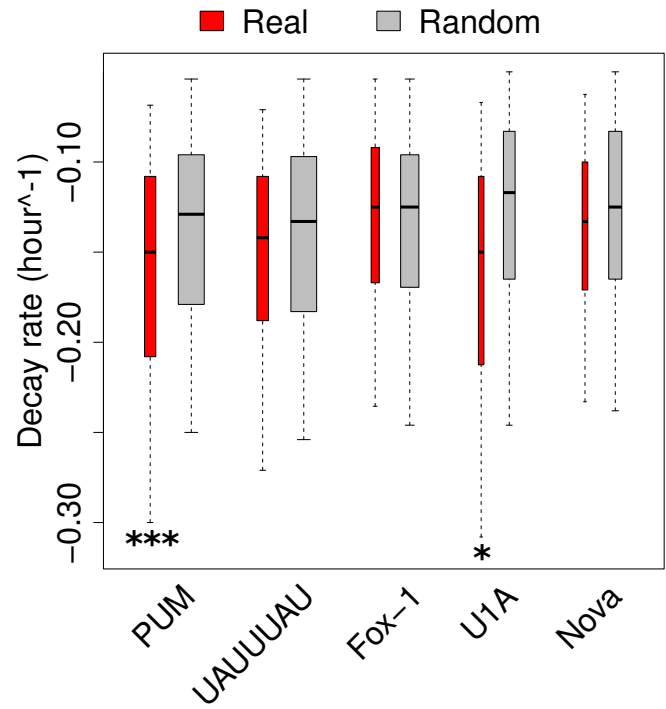

Supplement: Figure S9 — The presence of PUM and UAUUUAU results in faster transcript decay. Decay rates are shown in box-plots for transcripts with recognition sites for the designated RBP or shuffled RBP motifs. The Wilcoxon-test with a Bonferroni correction was applied to measure the difference between real RBP sites (Real) and shuffled RBP controls (Random). For each RBP, an asterisk designates a significant difference between transcripts with RBP recognition sites and transcripts with shuffled RBP motif sites. One asterisk indicates p<0.05, two asterisks indicate p<0.01, and three asterisks indicate p<0.001. (A, B) Half-lives are based on the published dataset [58]. (C) Decay rates are based on the published dataset [59]. (PDF) [file pcbi.1003075.s009.pdf]

Supplementary Figure S10

A. Decay rate

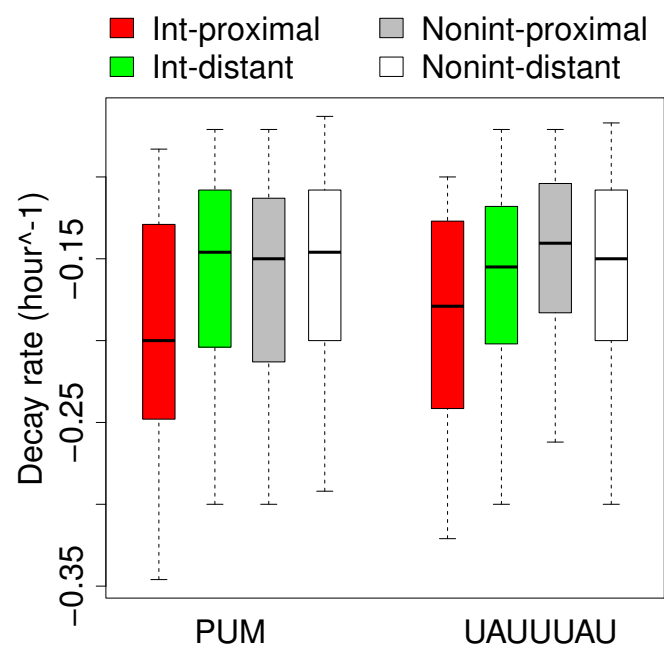

B. Primate conservation

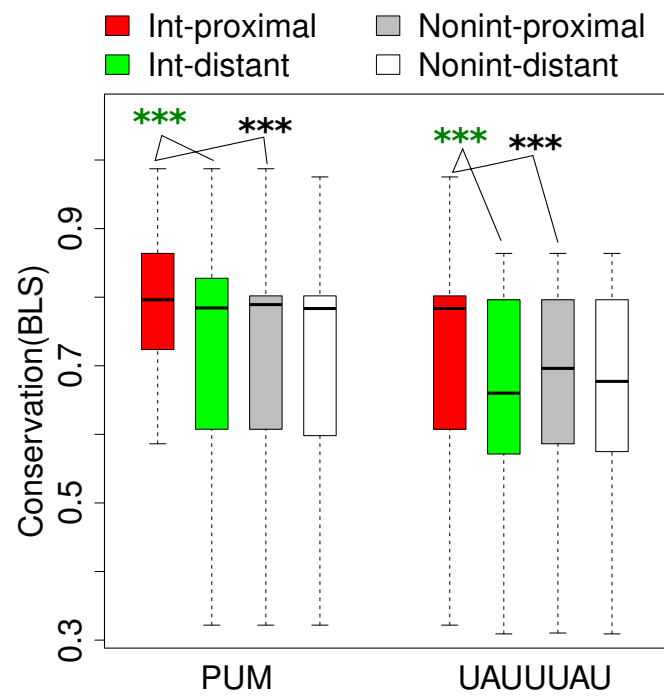

Supplement: Figure S10 — Pumilio recognition sites promote decay more effectively and are better conserved when present with interacting miRNAs. For each RBP, miRNAs were classified into four groups as described for Figure 4. (A) Decay rates were plotted based on dataset [59] as described in Figure 4A, B. (B) Conservation BLS scores were calculated based on ten primate species alignment, and plotted as described in Figure 4C, D. (PDF) [file pcbi.1003075.s010.pdf]

Supplementary Figure S11

A.Human

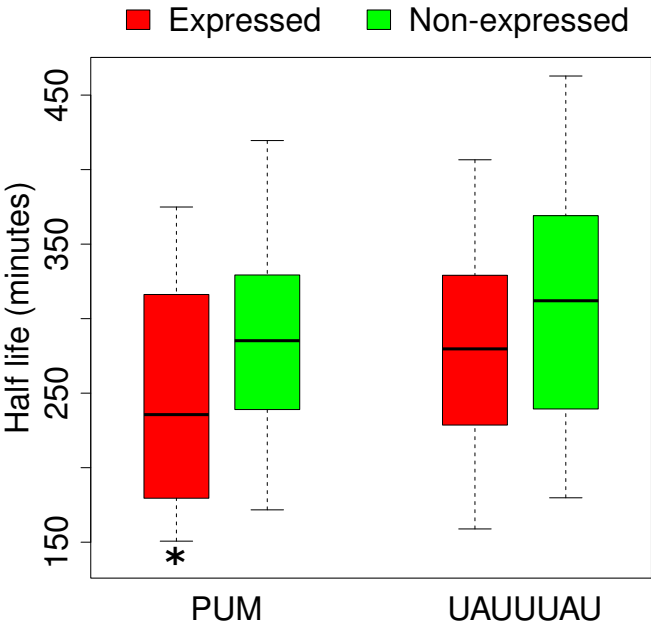

B.Mouse

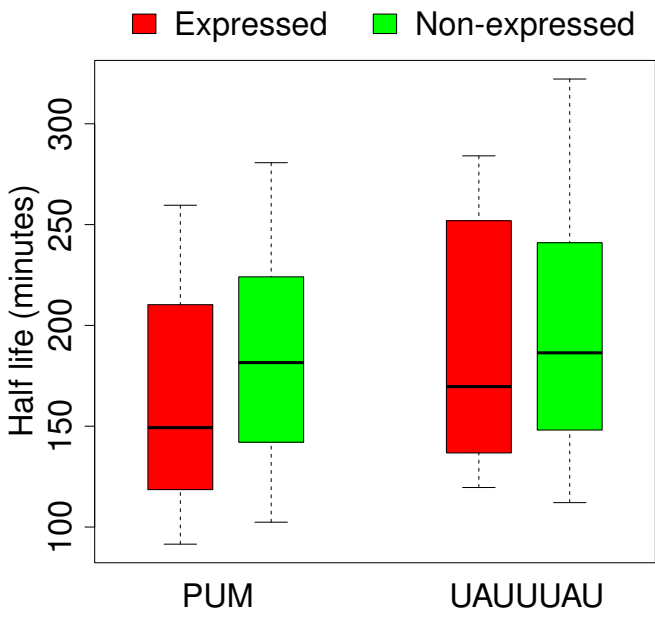

C.Human

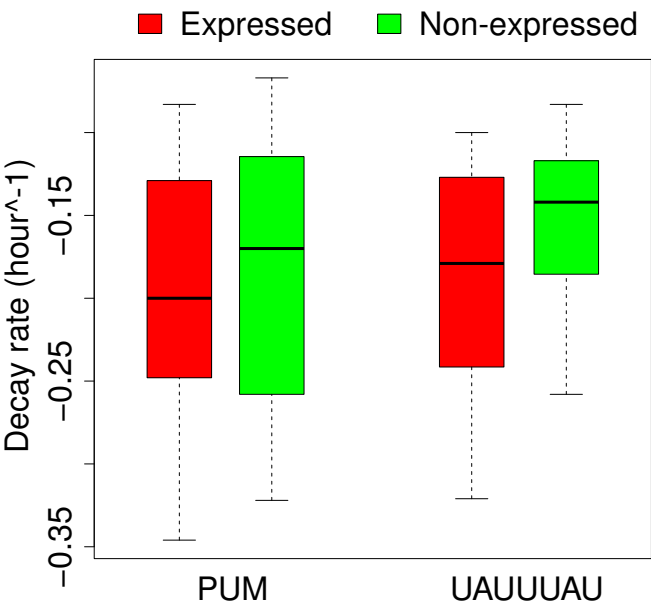

Supplement: Figure S11 — Expressed miRNAs promote decay more effectively than non-expressed miRNAs. For each of the cell lines used in half-life or decay rate datasets, companion small RNA sequencing datasets were identified from the literature. In each dataset, the reads of miRNAs were ranked and the most frequently expressed 25% of small RNA reads was established as a threshold for classifying interacting miRNAs for each RBP as “Expressed” or “Non-expressed”. Transcripts with proximal miRNA and RBP sites were compared with respect to their half-lives or decay rates. Asterisks represent comparisons of half-lives between two groups determined by Wilcoxon tests. One asterisk indicates p<0.05, two asterisks indicate p<0.01, and three asterisks indicate p<0.001. (A) For mRNA half-lives measured in Human B cells (BL41) [58], miRNA sequencing reads were derived from a dataset generated by Landgraf and colleagues [60]. (B) For mRNA half-lives measured in mouse fibroblasts (NIH-3T3) [58], miRNA sequencing reads were derived from dataset generated by Zhu and colleagues [61]. (C) For mRNA decay rates measured in human HepG2 [59], miRNA sequencing reads were derived from dataset generated by the ENCODE project [62]. (PDF) [file pcbi.1003075.s011.pdf]

Supplementary Figure S12

A.Human

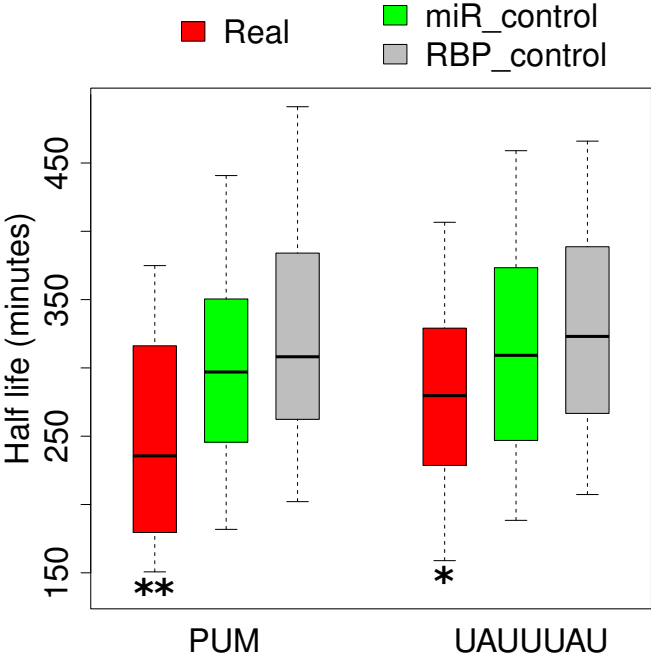

B.Mouse

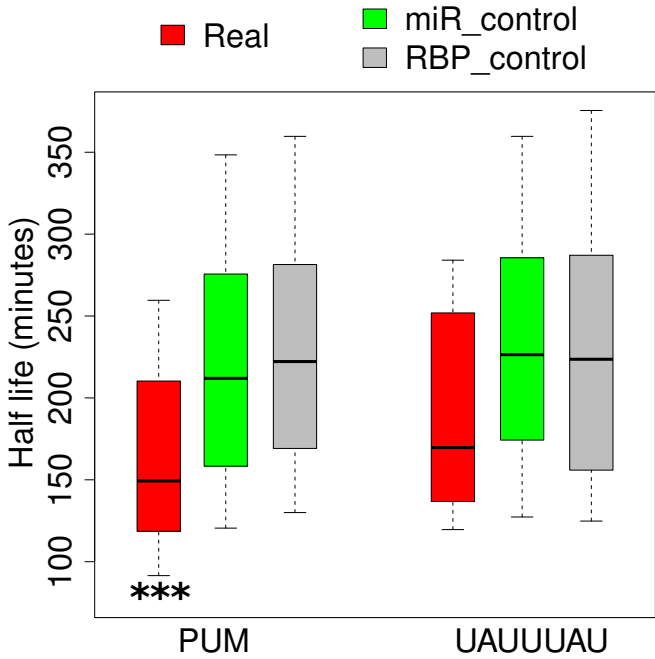

C.Human

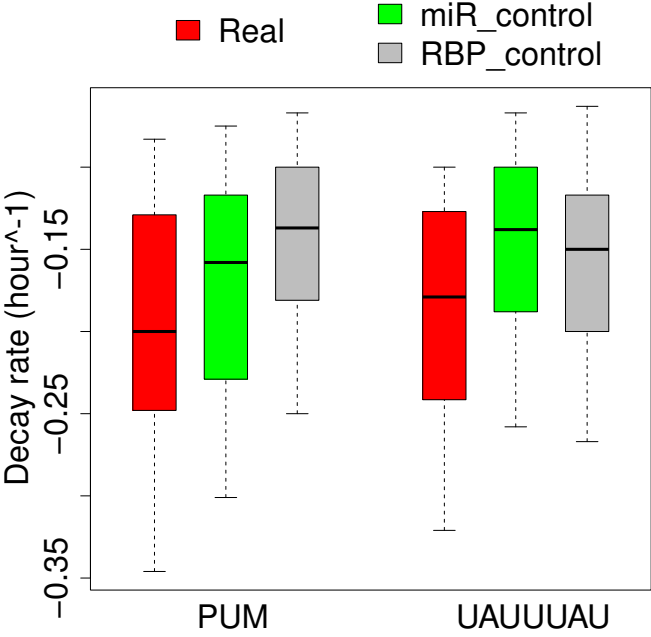

Supplement: Figure S12 — More rapid mRNA decay in transcripts in which Pumilio and interacting miRNA recognition sites colocalize is not only a consequence of AU-content. For each RBP or miRNA recognition motif, the shuffled RBP or miRNA motifs were used as controls for AU content. For each group of transcripts, boxplots of half-lives or decay rates were plotted as described for Figure 4A, B. Group “Real” contained transcripts with at least one RBP recognition site and a recognition site for one of the RBP's interacting miRNA within 50 nts. Group “miR control” contained transcripts with at least one RBP recognition site and a recognition site of shuffled interacting miRNA motif within 50 nts. Group “RBP control” contained transcripts with at least one recognition site of a shuffled RBP motif and an associated interacting miRNA recognition site within 50 nts. Asterisks represent comparisons of half-lives between the groups “Real” and “miR control” determined by Wilcoxon tests. One asterisk indicates p<0.05, two asterisks indicate p<0.01, and three asterisks indicate p<0.001. (A, B) Half-life data from Friedel and colleagues [58], (C) Decay rate data from Yang and colleagues [59]. (PDF) [file pcbi.1003075.s012.pdf]

Supplementary Figure S13

A. Human

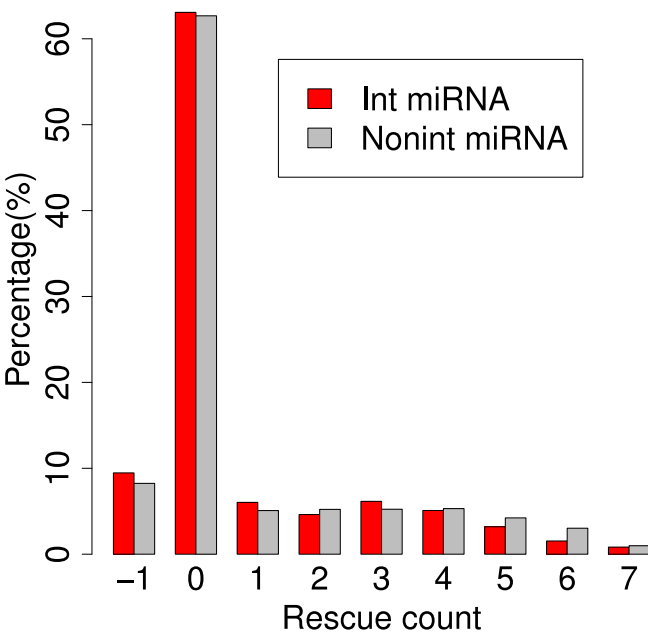

B. Mouse

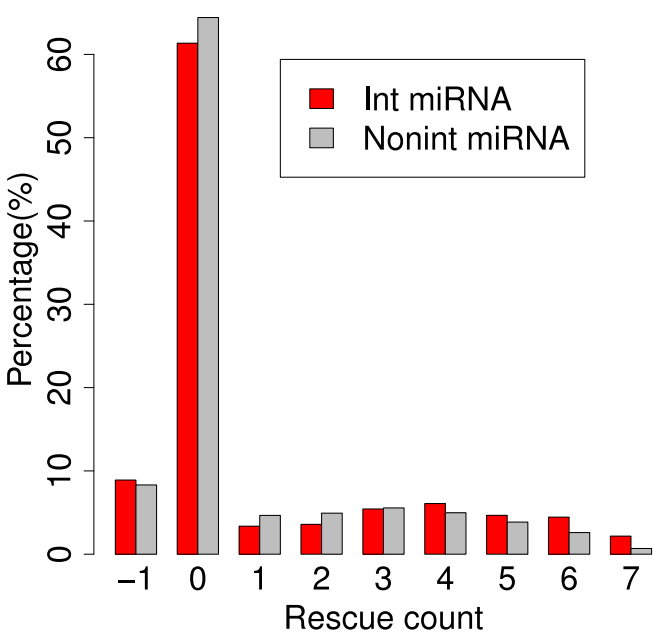

C. Human

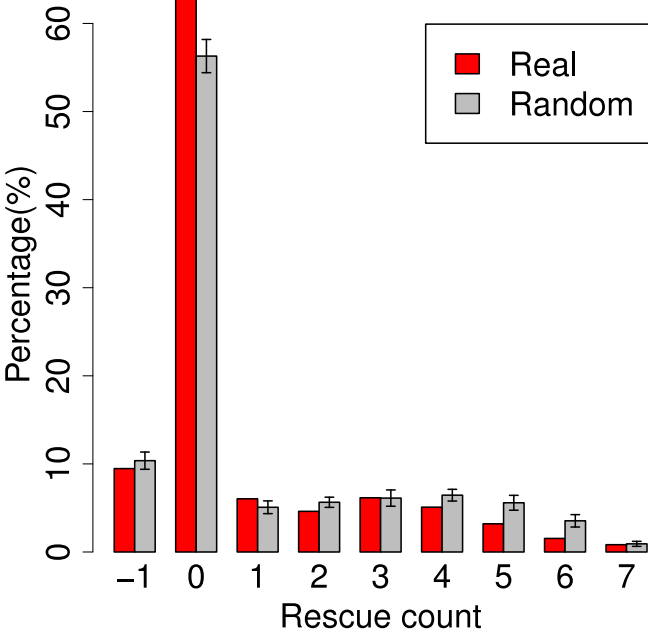

D. Mouse

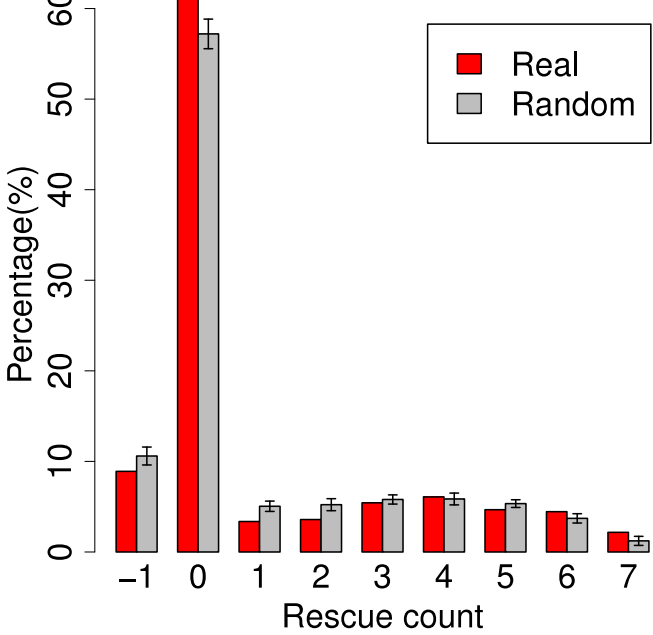

Supplement: Figure S13 — Histograms of rescue counts for miRNA recognition sites upon UAUUUAU binding. Histograms of site rescue were calculated for both UAUUUAU-interacting miRNAs and non-interacting miRNAs as described in Figure 5. (A, B) Comparison of histograms between interacting miRNAs and non-interacting miRNAs are shown separately for human and mouse. (C, D) Comparison of histograms between real rescue counts and random rescue counts determined based on a background model are shown for human and mouse. (PDF) [file pcbi.1003075.s013.pdf]

Supplementary Figure S14

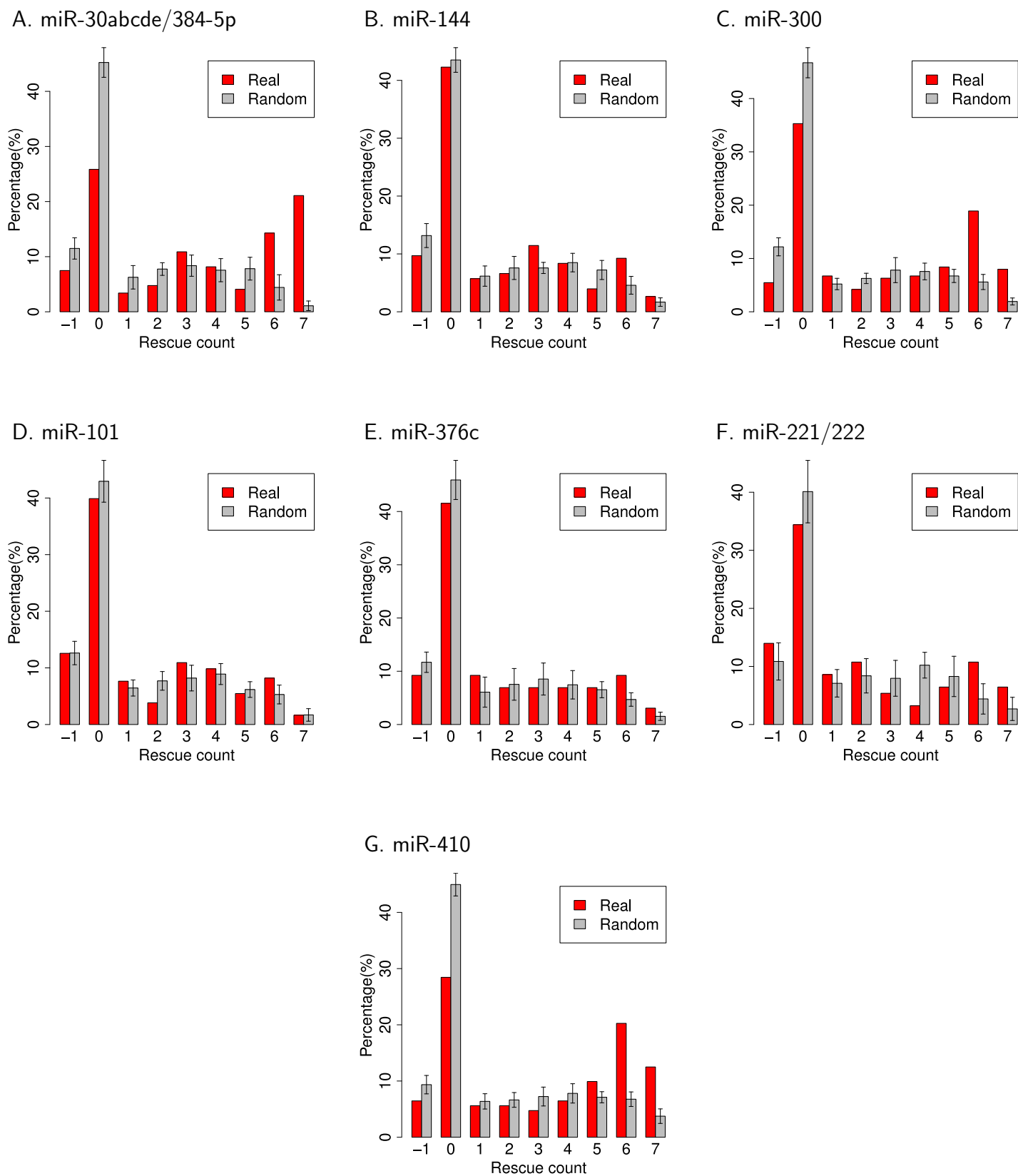

Supplement: Figure S14 — PUM rescues nucleotides in neighboring interacting miRNA recognition sites. The histogram of miRNA seed rescue was compared with rescue values calculated with the background model of Figure 5C, D. Data for each of the seven interacting miRNAs of PUM are shown separately for the human genome. (A) miR-30abcde/384-5p. (B) miR-144. (C) miR-300. (D) miR-101. (E) miR-376c. (F) miR-221/222. (G) miR-410. (PDF) [file pcbi.1003075.s014.pdf]

Supplementary Figure S15

A. Human

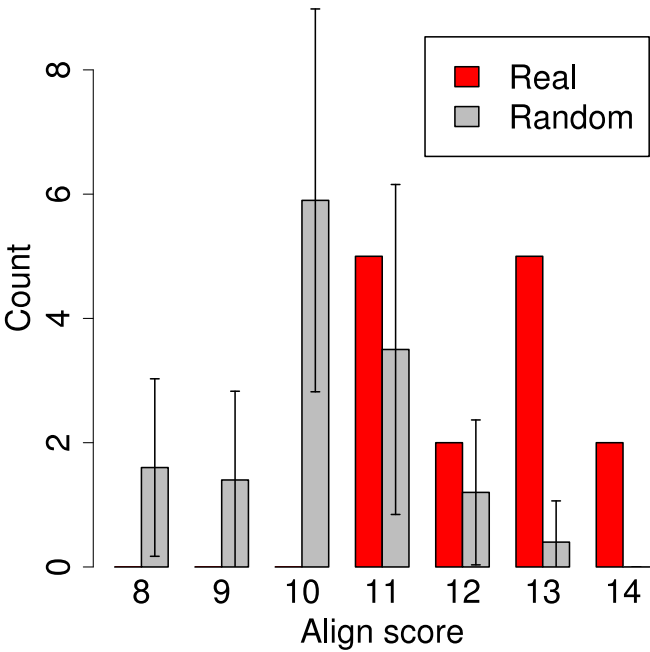

B. Mouse

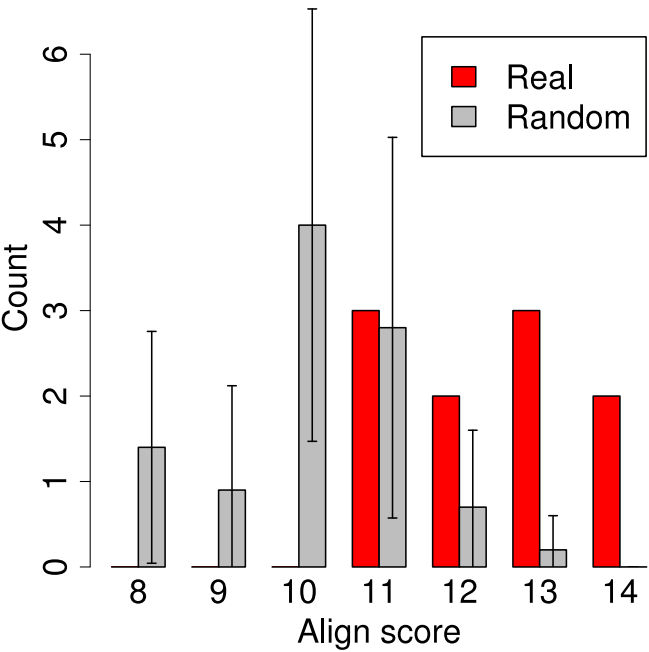

Supplement: Figure S15 — Histograms of alignment scores between miRNA seeds and Pumilio recognition motifs. For all interacting miRNAs of PUM, their seed alignment scores with the reverse PUM motif were determined for real PUM motif and shuffled PUM motifs. Histograms for all shuffled PUM motifs were merged into average values and standard deviations, and are shown for PUM interacting miRNAs identified in (A) Human and (B) Mouse. (PDF) [file pcbi.1003075.s015.pdf]
