# Supplementary material for: Computational Assessment of the Cooperativity between RNA Binding Proteins and MicroRNAs in Transcript Decay
Source: PLoS Comput Biol. 2013 May 30;9(5):e1003075. doi: 10.1371/journal.pcbi.1003075 (PMC3667768; doi:10.1371/journal.pcbi.1003075)
Supplement: Table S2 — AU composition of RBP recognition motifs and interacting miRNA recognition seeds. The fraction of nucleotides that are AU was calculated for each RBP recognition motif and the recognition seeds of miRNAs that interact with each RBP. The average value is shown. The average AU fraction for all miRNA seeds is 52.7%. (PDF) [file pcbi.1003075.s017.pdf]

Supplementary Table S2

RBP	Recogintion motif	Interacting miRNA seeds
PUM	81.3%	69.4%
UAUUUAU	100%	71.4%
