# Supplementary material for: Computational Assessment of the Cooperativity between RNA Binding Proteins and MicroRNAs in Transcript Decay
Source: PLoS Comput Biol. 2013 May 30;9(5):e1003075. doi: 10.1371/journal.pcbi.1003075 (PMC3667768; doi:10.1371/journal.pcbi.1003075)
Supplement: Table S3 — miRNA sites are enriched around RBP sites in human and mouse 3′UTRs. For each organism, the ratio of miRNA recognition site density 50 nts upstream or downstream of the RBP recognition sites to miRNA site density across all 3′UTRs is reported for PUM and UAUUUAU. (PDF) [file pcbi.1003075.s018.pdf]

Supplementary Table S3

	PUM	UAUUUAU
H.sapiens	1.12	1.14
M.musculus	1.12	1.06
D.melanogaster	0.87	0.95
C.elegans	1.07	0.87
