# Supplementary material for: Computational Assessment of the Cooperativity between RNA Binding Proteins and MicroRNAs in Transcript Decay
Source: PLoS Comput Biol. 2013 May 30;9(5):e1003075. doi: 10.1371/journal.pcbi.1003075 (PMC3667768; doi:10.1371/journal.pcbi.1003075)
Supplement: Table S4 — Expressed RBP-interacting miRNAs and their effects on mRNA decay. For each of the mRNA decay datasets, we considered each RBP and its expressed interacting miRNAs (corresponding to Figure 4A, B). Proximal and distant pairs of recognition sites were defined as in Figure 4. The median mRNA half-lives or decay rates were shown for each combination. P-values were calculated from Wilcoxon rank test as a measure of the difference between proximal and distant group. (A) Results from mRNA half-life datasets of human B cells [58]. (B) Results from mRNA half-life datasets of mouse fibroblasts [58]. (C) Results from mRNA decay rate dataset of HepG2 cell line [59]. (PDF) [file pcbi.1003075.s019.pdf]

Supplementary Table S4

RBP	miRNA	Half life (minutes)		Number of mRNAs		P-value
		Proximal	Distant	Proximal	Distant	
PUM	miR-30abcde/384-5p	214.7	302.1	55	605	1.21E-2
	miR-101	254.6	300.2	88	585	9.06E-3
UAUUUAU	miR-30abcde/384-5p	262.4	304.8	69	691	2.78E-3
	miR-26ab/1297	309.0	310.3	64	696	4.29E-1

(a) Human Friedel

RBP	miRNA	Half life (minutes)		Number of mRNAs		P-value
		Proximal	Distant	Proximal	Distant	
PUM	miR-30abcde/384-5p	138.8	187.1	57	539	5.76E-2
	miR-101	153.3	195.9	72	557	4.03E-2
UAUUUAU	miR-26ab/1297	169.65	208.0	64	565	2.18E-1

(b) Mouse Friedel

RBP	miRNA	Decay rate (hourˆ1)		Number of mRNAs		P-value
		Proximal	Distant	Proximal	Distant	
PUM	miR-410	-0.221	-0.15	48	273	1.45E-2
	miR-376c	-0.22625	-0.15	32	165	9.95E-2
	miR-30abcde/384-5p	-0.146	-0.179	21	251	2.50E-1
	miR-101	-0.1405	-0.15	34	241	4.08E-1
UAUUUAU	miR-30abcde/384-5p	-0.233	-0.163	21	271	3.20E-1
	miR-26ab/1297	-0.179	-0.15	25	286	1.82E-1

(c) Human Yang
