# Supplementary material for: Computational Assessment of the Cooperativity between RNA Binding Proteins and MicroRNAs in Transcript Decay
Source: PLoS Comput Biol. 2013 May 30;9(5):e1003075. doi: 10.1371/journal.pcbi.1003075 (PMC3667768; doi:10.1371/journal.pcbi.1003075)
Supplement: Table S5 — RBP recognition sites are more conserved when present with interacting miRNAs. For each RBP and its interacting miRNAs, their neighbor recognition sites were classified into either the proximal or distant groups as described for Figure 4. The median conservation BLS scores are shown for each combination and p-values are calculated based on Wilcoxon rank tests as a measure of the difference between the two groups. (A) Human-interacting miRNAs are shown. (B) Mouse-interacting miRNAs are shown. (PDF) [file pcbi.1003075.s020.pdf]

Supplementary Table S5

RBP	miRNA	Conservation BLS		Number of RBP sites		P-value
		Proximal	Distant	Proximal	Distant	
PUM	miR-30abcde/384-5p	0.686	0.630	147	1773	7.00E-2
	miR-144	0.702	0.649	227	2003	7.12E-3
	miR-300	0.767	0.633	236	2299	9.03E-6
	miR-101	0.712	0.657	183	1765	7.38E-4
	miR-376c	0.706	0.607	130	1404	3.01E-3
	miR-221/222	0.722	0.646	92	1212	2.41E-2
	miR-410	0.654	0.604	230	1971	2.74E-2
UAUUUAU	miR-30abcde/384-5p	0.612	0.462	208	2258	1.39E-4
	miR-144	0.626	0.475	215	2365	1.42E-5
	miR-494	0.511	0.453	244	2865	2.81E-1
	miR-26ab/1297	0.485	0.443	167	2188	2.26E-1

(a) Human

RBP	miRNA	Conservation BLS		Number of RBP sites		P-value
		Proximal	Distant	Proximal	Distant	
PUM	miR-30abcde/384-5p	0.297	0.262	146	1450	4.18E-1
	miR-144	0.338	0.252	180	1586	1.80E-3
	miR-300	0.336	0.247	222	1800	3.13E-4
	miR-101	0.354	0.266	153	1526	2.57E-3
	miR-376c	0.253	0.207	107	1084	1.43E-1
UAUUUAU	miR-300	0.257	0.183	189	1770	1.75E-4
	miR-494	0.203	0.166	165	1812	6.21E-2
	miR-26ab/1297	0.181	0.165	141	1533	1.02E-1
	miR-181	0.271	0.174	152	1653	1.74E-5
	miR-495/1192	0.198	0.176	272	2210	3.45E-1

(b) Mouse
