# Supplementary material for: Computational Assessment of the Cooperativity between RNA Binding Proteins and MicroRNAs in Transcript Decay
Source: PLoS Comput Biol. 2013 May 30;9(5):e1003075. doi: 10.1371/journal.pcbi.1003075 (PMC3667768; doi:10.1371/journal.pcbi.1003075)
Supplement: Table S6 — GO enrichments for PUM and its interacting miRNAs. Transcripts were classified into categories as in Figure 4. The GO biological process annotations were compared between group “Int-proximal” (transcripts with at least one RBP site and its interacting miRNA recognition site within 50 nts) and group “Int-distant” (transcripts with both RBP sites and its interacting miRNA recognition sites, but no pair of recognition sites is within 50 nts). Hypergeometric enrichment was used to calculate p-values. We then applied the Benjamini-Hochberg procedure on the p-values, and selected enriched GO terms with FDR< = 0.05. The number of annotated genes and hypergeometric p-values are shown for each significant GO term. (PDF) [file pcbi.1003075.s021.pdf]

# Supplementary Table S6

| GO Biological process | #genes | P-value |
|---|---|---|
| protein binding transcription factor activity | 38 | 4.09E-4 |
| transcription factor binding transcription factor activity | 37 | 5.36E-4 |
| transcription cofactor activity | 36 | 5.39E-4 |
